# Person-centered shared decision-making and data-informed district nursing care to enhance independence: Protocol for a feasibility study

**DOI:** 10.1016/j.ijnsa.2026.100569

**Published:** 2026-06-01

**Authors:** Sigrid Wulfse-Huisman, Xenia Yocarini, Jessica Veldhuizen, Koen van den Braak, Mariëlle Zondervan-Zwijnenburg, Ruth Pel-Littel, Bianca Buurman, Nienke Bleijenberg

**Affiliations:** a Department of Internal Medicine, Section of Geriatrics and Amsterdam Public Health, Academic Medical Center, University of Amsterdam, Meibergdreef 9, 1105 AZ Amsterdam, the Netherlands; b Amstelring District Nurse Care, Laan van de Helende Meesters 431, 1186 DK Amstelveen, the Netherlands; c Research Group Proactive care for older people living at home, Research Center for Healthy and Sustainable Living, University of Applied Sciences, Heidelberglaan 7, 3501 AA Utrecht, the Netherlands; d Vilans, Center of Expertise for Long-term Care, Postbus 8228, 3503 RE Utrecht, the Netherlands; e HAN University of Applied Sciences, Department of Health and Vitality, Kapittelweg 33, 6525 EN Nijmegen, the Netherlands; f VU University Medical Center, Department of Geriatrics, De Boelelaan 1109, 1081 HV Amsterdam, the Netherlands; g Department of General Practice & Nursing Science, Division Julius Center for Health Sciences and Primary Care, University Medical Center Utrecht, Universiteitsweg 100, 3584 CG Utrecht, the Netherlands

**Keywords:** Aged, Shared decision-making, Feasibility studies, Independent functioning, Home health nursing, Nursing informatics, Dashboard

## Abstract

**Background:**

Maintaining independence is a key priority for older adults and a central goal of district nursing care. Although person-centered shared decision-making and the use of routinely collected care data are recognized as strategies to support independence, their combined use in district nursing care has not yet been studied.

**Objective:**

This study has two aims. The primary aim is to assess the feasibility, acceptability, fidelity, experiences, and barriers and facilitators of the Data Nurse intervention among district nursing teams and older adults. The secondary aim is to explore preliminary effectiveness on older adults' independent functioning and their perceived shared decision-making.

**Design and setting:**

A multicenter non-randomized feasibility study with an intervention group and a control group. Four district nursing organizations in the Netherlands will participate, representing urban and rural areas.

**Participants:**

A total of 14 district nursing teams, with eight intervention and six control teams, with approximately 84–109 district nursing professionals and 210–294 older adult participants will be included.

**Methods:**

The Data Nurse intervention combines two components: (1) person-centered shared decision-making with older adults, supported by a patient preparatory tool shared decision-making, and (2) learning from data via a digital dashboard that visualizes longitudinal independence data from electronic nursing records. In this continuous cycle, interventions to support independent functioning are planned and documented; independence data are visualized and discussed during team reflection sessions, discussed with patients, and interventions to support independent functioning are evaluated and adjusted as needed. Primary outcomes on feasibility, acceptability, fidelity, experiences, barriers and facilitators will be assessed through the validated questionnaires Feasibility of Intervention Measure, the Theoretical Framework of Acceptability questionnaire, the Theoretical Domains Framework, self-developed questionnaires, focus groups, and interviews with patients and district nurses. Fidelity will be monitored through monthly meetings using a self- developed questionnaire. Secondary outcomes on preliminary effectiveness on independent functioning will be measured using selected domains of the TOPIC-SF questionnaire and electronic nursing record data. In addition, patient perceived involvement in the shared decision-making process is measured with the CollaboRATE. Data will be analyzed using descriptive statistics and thematic analysis.

**Results:**

No results are available at the time of submission.

**Conclusions:**

This protocol describes a feasibility study to generate evidence on the feasibility and preliminary effectiveness of a novel complex intervention combining person-centered shared decision-making with data-informed care in district nursing to enhance independence. Findings will inform the intervention refinement.

**Registration:**

Trial registration: ISRCTN, 14972807, registered [8 December 2025], first participant recruited [4 June 2025].

**Social media abstract:**

Testing the Data Nurse intervention: combining care data & shared decision-making to support independence in older adults across 14 Dutch district nursing teams.

**What is already known?**

• Maintaining independent functioning is a key priority for older adults and a central objective in district nursing care, yet effective strategies to support this in this setting remain scarce.
• Older adults receiving district nursing care are often unaware that collaborative goal-setting for their care is possible, which limits their active involvement in decision-making.
• Although electronic nursing records capture longitudinal care data, this information is rarely used to monitor trends in independent functioning or to inform person-centered shared decision-making with older adults.

Alt-text: Unlabelled box dummy alt text

**What this paper adds**

• The Data Nurse intervention is the first to systematically combine person-centered shared decision-making with data-informed care in a continuous cycle to support independence of older adults with district nursing care.
• This protocol describes the design and methods for a multicenter feasibility study that will generate evidence on feasibility and preliminary effectiveness of this novel complex intervention across 14 Dutch district nursing teams.

Alt-text: Unlabelled box dummy alt text

## 1 Background

Worldwide, older adults want to stay in their own homes as they age, valuing independence and safety. Although aging often involves multiple chronic conditions and independent functioning related problems, the desire to live independently remains strong (Chowdhury et al., 2023). District nursing care supports the goal of older adults aging in place by making person-centered shared decisions about care and interventions that help maintain daily functioning and delay or prevent institutionalization (Dostálová et al., 2021). The growing number of older adults with complex care needs is placing increasing demands on district nursing care (McGilton et al., 2018). Beyond illness-related care needs, these older adults have specific physical, psychological, and social needs related to maintaining their independent functioning (Ho et al., 2023; Marriott-Statham et al., 2023). This has intensified the need for district nursing interventions that actively support and sustain independence in daily life (Hoffmann et al., 2018).

In line with the Dutch Health Council, in this study, independent functioning for home-dwelling older adults is described as the capacity to maintain physical, psychological, and social wellbeing, with or without formal or informal care, and to retain control over daily life during a stage marked by inevitable changes and losses (Gezondheidsraad, 2018; Huber et al., 2011). Supporting independent functioning requires two core elements that together form the focus of this study. The first is person-centered shared decision-making, defined as a collaborative process in which healthcare decisions are made jointly by patients, their families, and healthcare professionals, with a primary focus on eliciting and prioritizing personal and care-related goals, and on aligning these goals with interventions (Elwyn and Vermunt, 2020). In contrast to disease-oriented shared decision-making, person-centered shared decision-making typically centres on the person across life domains and uses their fundamental goals, values, and priorities as the basis for care decisions, instead of focusing solely on biomedical abnormalities, the medical management of underlying conditions, or adhering to clinical guidelines designed for single diseases (Elwyn and Vermunt, 2020; Van de Pol et al., 2016). The second is data-informed care, which we define as the structured use of routinely collected patient data, including longitudinal information on functioning, to support clinical reasoning and shared decisions about care (Veldhuizen et al., 2024a; Veldhuizen et al., 2024b).

Two critical gaps exist in current district nursing practice. First, although shared decision-making is legally mandated and widely promoted in Dutch healthcare policy (Government of the Netherlands, 2023), its implementation in everyday district nursing practice (Marriott-Statham et al., 2023) and education (Noordam et al., 2024), remains limited. A recent review (Mills et al., 2025) found that although district nurses and patients reported valuing collaborative decision-making, the process is not consistently embedded in practice, with wide variation in how and whether nurses involve patients in care decisions. A recent video observation study of real-world district nursing conversations, during intakes and evaluations, showed that steps from person-centered models for shared decision-making were largely absent: preparation for the conversation was not offered to older adults, conversations lacked goal-setting, and independence was primarily addressed in relation to the referrer's care request rather than holistically across life domains (Wulfse-Huisman et al., 2025b). Moreover, older adults appeared unaware that shared decision-making about independence was possible within district nursing care, leaving their goals and concerns in health and social domains undiscussed (Wulfse-Huisman et al., 2025b; Wulfse-Huisman et al., 2025a). While structured person-centered shared decision-making models exist for hospitals and general practices (Elwyn and Vermunt, 2020; van de Pol et al., 2016), no equivalent models have been developed for or evaluated in district nursing care.

Second, data in electronic district nursing records is primarily used for documentation purposes rather than to support care decisions (Veldhuizen et al., 2024a). A systematic review of digital health systems in nursing found that while assessment and planning are increasingly captured electronically, the documentation and evaluation of nursing interventions and patient outcomes remain largely absent, limiting nurses' ability to use data for decision-making (Hants et al., 2023). A recent scoping review of shared decision-making tools integrated into electronic health records similarly found that most tools were designed primarily for clinicians, with patient-specific goals and values included in only just over half of the studies and that integration approaches varied widely: from scanned paper documents to fully interoperable tools (Pierce et al., 2025). This is particularly relevant for district nursing care, where care planning must align with older adults' daily lives and long-term needs. Electronic nursing records contain valuable information on care delivery patterns. However, this data is rarely used to monitor longitudinal trends in independent functioning or to inform collaborative decision making with older adults (Siette et al., 2023). The potential of using electronic nursing record data to substantiate and evaluate nursing interventions that support independence thus remains untapped.

While both person-centered shared decision-making and data-informed care are individually recognized as important strategies to enhance care quality and patient independence, their combined implementation in district nursing care has not yet been studied. In response to these knowledge gaps, we developed the Data Nurse intervention, which combines person-centered shared decision-making with data-informed care for supporting older adults' independence. This article describes the protocol for a multicenter feasibility study of the Data Nurse intervention.

## 2 Aims

The aim of the feasibility study is twofold:

Primary aims:

• To assess the feasibility, acceptability, fidelity, barriers, facilitators, and experiences of using the Data Nurse intervention among members of district nursing teams and older adults receiving district nursing care.

Secondary aims:

• To explore the preliminary effectiveness of the intervention on older adults' (self-reported) independence and older adults’ self-reported perceived shared decision-making.

## 3 Methods

### 3.1 Study design

This study is designed as a pragmatic, non-randomized multicenter feasibility study with an intervention and a control group, following a mixed-method approach. This study is developed in line with the updated guidance for feasibility studies by the MRC framework (Skivington et al., 2024) and checklist for developing and evaluating complex interventions (Skivington et al., 2024) (Supplementary File 1).

### 3.2 Context

In this paper, the description of district nursing care is described according to the “Expertise Area of District Nursing Care”, as outlined by the Dutch Nursing Association (V&VN): “Care for all residents in their own living environment, focused on promoting health, independent functioning, and providing nursing and personal care, behind the front door and in the community, with an emphasis on person-centered shared decision-making and interprofessional collaboration, enabling individuals to live at home for as long as possible. It is a broad role that includes both curative care and prevention, with the district nursing teams being the central professionals in connecting care, housing, and wellbeing" (Rosendal, 2019). In the Netherlands, district nursing care is delivered to over half a million people, with >80% aged 65 or older (Vektis, 2025). District nursing care is provided by autonomous teams consisting of professionals ranging from healthcare assistants (European Qualifications Framework level 2), nursing assistants (European Qualifications Framework level 3), vocational nurses (European Qualifications Framework level 4), and district nurses (European Qualifications Framework level 6) to nurse practitioners (European Qualifications Framework level 7). District nurses act as gatekeepers, assessing and determining the type, intensity, and duration of district nursing care (Schwenke et al., 2023). Since January 2020, shared decision-making in healthcare has been legally required in the Netherlands (National Health Care Institute, n.d.).

### 3.3 Study setting

This study will be conducted in the Netherlands as part of the Data Nurse project, titled “Data-informed Essential Care in District Nursing Care: Improving Patient Outcomes and Maintaining Independence”. Four large care organizations that provide district nursing care across the Netherlands, selected to represent both urban and rural areas, will participate. Participants include district nursing team members and older adults aged 65 and older who receive district nursing care.

### 3.4 Intervention development

**Fig. 1:**
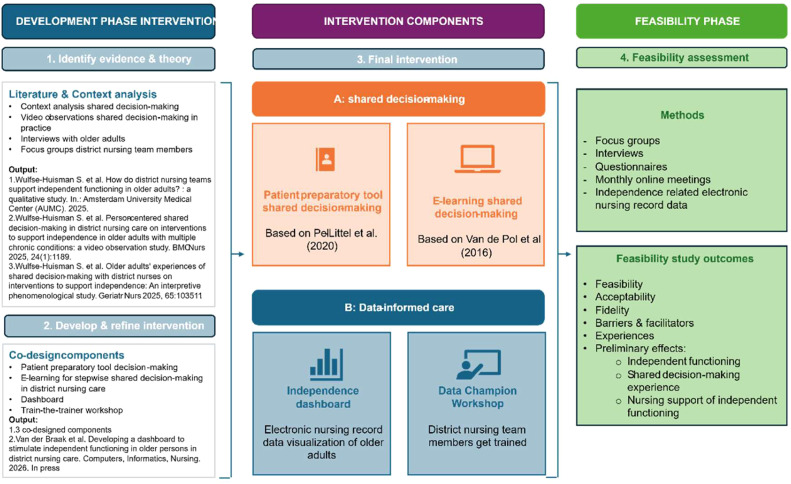
Intervention development.

The Data Nurse intervention was developed through systematic phases of exploration, co-design, and refinement in close collaboration with district nurses and older adults. Fig. 1. presents an overview of the development steps.

The development process involved the following sequential steps:

1) Identifying gaps in the literature
  The initial stage of the development process involved a comprehensive review of the existing literature to identify gaps in evidence and guidance. Based on these gaps, the context of district nursing care was explored.
2) Understanding the needs of the context
  An exploration of older adults' experiences with shared decision-making in district nursing care was conducted through qualitative research (Wulfse-Huisman et al., 2025a). This study identified the needs of older adults to meaningfully participate in the decision-making process with district nursing professionals.
3) Exploring current practice
  The context and current practices of person-centered shared decision-making on interventions to support independence in district nursing were systematically explored (Wulfse-Huisman, 2025c; Wulfse-Huisman et al., 2025b).
4) Co-designing Component 1 of the Data Nurse intervention - Shared Decision-Making
  Based on insights from Steps 1,2 and 3, two tools (a and b) were co-designed:
  a) **Patient preparatory tool shared decision-making**
    An existing preparatory tool for older adults from geriatric wards and general practices (Pel-Littel et al., 2020) was adapted through co-design with older adults receiving district nursing care and district nursing team members. Fifteen older adults completed this preparatory tool at home and provided feedback on its feasibility using a questionnaire. Five district nurses discussed the preparatory tool with these older adults and indicated its feasibility via a questionnaire. Two researchers (RPL, SWH) then used the answers from the completed questionnaires to make adjustments to the existing preparatory tool. Two adjustments were made: (1) the question about hearing problems was added and (2) the question about vision problems was added.
  b) **E-learning Program for stepwise shared decision-making in District Nursing care**
    A structured e-learning module was developed based on an existing stepwise person-centered shared decision-making conversation model for healthcare professionals(van de Pol et al., 2016). The program was adapted to the specific needs and context of district nursing practice through consultation with nursing educators, practicing district nurses, and shared decision-making experts (Dialogue Trainer, 2025). The e-learning was pilot-tested with five district nurses and refined on their feedback. Based on this feedback, a scenario was created featuring a virtual patient who does not immediately understand that district nursing professionals do not automatically take over care but rather support independence. Supplementary File 2. shows the English translation of the patient preparatory tool shared decision-making, the person-centered shared decision-making conversation model, and link to the free e-learning from Dialogue Trainer (Dutch).
5) Co-designing Component 2 of the Data Nurse intervention- learning from data via dashboard
  A digital dashboard visualizing independence-related data was co-designed and iteratively tested with district nursing teams using a user-centered design approach (van den Braak et al., 2026). Multiple prototypes were refined based on nurses’ feedback regarding usability, data relevance, visual clarity, alignment with the nursing process, and workflow integration. Developed using Qlik Cloud, the dashboard displays longitudinal trends in patients’ independent functioning and nursing interventions, structured according to the Omaha System, and links registered problems to intervention targets and practical aids. The dashboard prototype was technically implemented by business intelligence experts from the participating organizations, who adapted the prototype to their existing data infrastructures and established a real-time connection with routinely registered electronic nursing record data.
6) Developing implementation support of the independence dashboard
  A train-the-trainer workshop was developed to support dashboard implementation in district nursing teams. The curriculum, designed in consultation with implementation science experts, was iteratively refined based on participant feedback. Six on-site workshops were conducted, targeting designated team leaders and data ambassadors, five with single teams (two participants each) and one with three teams jointly (six participants). The workshops prepared participants to train colleagues in dashboard use and facilitate team-based reflection on care practices, integrating the tool into routine meetings and case discussions to support a structured learning and improvement cycle. Appendix 1. Shows this cycle.
7) Integration and finalization of the components of the Data Nurse intervention
  The two components of the Data Nurse intervention were integrated into a coherent package. The logic model shows the sequence in which the intervention components were used.

### 3.5 The data nurse intervention

The Data Nurse intervention integrates person-centered shared decision-making with data-informed reflective practice in a continuous cycle in clinical practice. In this cycle, older adults are encouraged and prepared to actively participate in shared decision-making by using the patient shared decision‑making preparatory tool to articulate and reflect on their independence goals, values, and care preferences through structured questions about daily functioning and what matters most to them (Supplementary File 2). Based on this tool, district nurses engage in shared decision-making conversations with older adults about interventions to support their independence. Care decisions and interventions are subsequently documented in the electronic nursing record using the Omaha System classification, generating data on nursing care delivery and patient functioning. This data will be used in the Data Nurse dashboard, visualizing longitudinal trends in patient independence scores across multiple domains (e.g., mobility, self-care, social functioning), frequency and types of nursing interventions aimed at supporting independence, individual patient trajectories, and team-level patterns in care delivery (van den Braak et al., 2026). In regular team reflection sessions, led by trained data, teams will review dashboard insights to identify patterns in the independent functioning of older adults, and evaluate interventions to support independence. These insights into independence will then be shared with older adults during care evaluations. This cycle of decision-making, documentation, visualization, and reflection is intended to close the loop between data and practice, supporting continuous learning and adaptation in daily care in line with the principles of a learning health system (Foley and Vale, 2023).

The intervention will be discontinued or modified for individual participants or teams in the following circumstances: participant withdrawal of consent, prolonged illness or incapacity of the participant, dissolution of the participating nursing team, or absence of a trained data champion for >4 consecutive weeks. In such cases, participants will continue to receive standard care. During the study period, participants are permitted to receive all standard district nursing care and other routine healthcare services. No co-interventions are explicitly prohibited, provided they are not part of a concurrent structured intervention targeting shared decision-making or data-informed nursing practice. Any co-interventions received will be recorded and reported as part of the study documentation.

### 3.6 Logic model

**Fig. 2:**
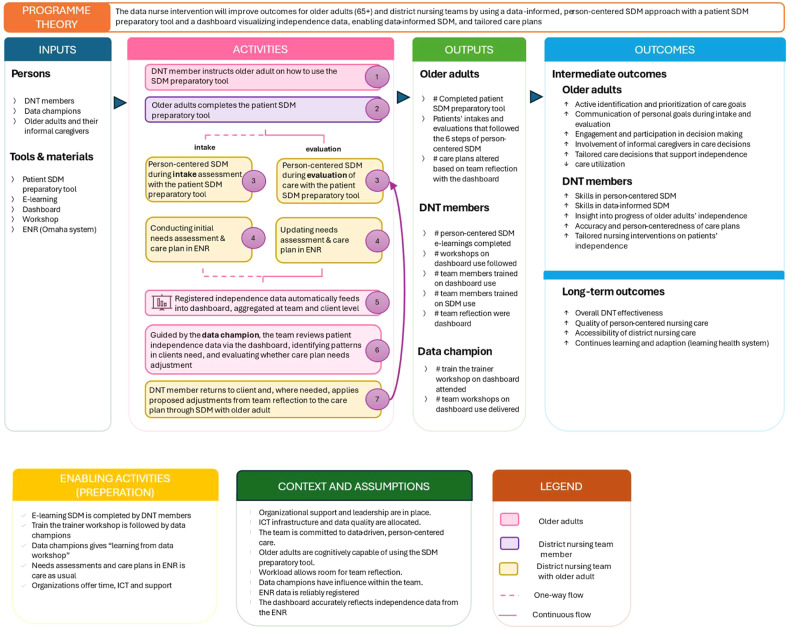
Logic model data nurse intervention. Abbreviations: DNT: District nursing team, SDM: Shared decision-making, ENR: Electronic nursing record.

Fig. 2. presents the logic model of the Data Nurse intervention, illustrating how the intervention components interact and explaining the possible working mechanism of these components together, which contribute to the expected/potential intervention effectiveness. It maps the relationships between inputs, activities, outputs, and anticipated outcomes, and identifies the contextual factors that may influence implementation and effectiveness (Funnell and Rogers, 2011).

#### 3.6.1 Training and preparing team members

Prior to implementation, district nursing teams will complete preparatory training for both components. For the shared decision-making component, team members will complete a structured e-learning module (Dialogue Trainer, 2025) of approximately 45 min, covering the steps of person-centered shared decision-making(van de Pol et al., 2016), documentation of patient preferences in the electronic nursing record, and scenario-based exercises for integrating shared decision-making into routine conversations (Supplementary File 2.). For the learning from data component, each intervention team will select one data champion based on voluntary interest and affinity with data use. These data champions will participate in a half-day train-the-trainer workshop covering dashboard navigation, data interpretation, and facilitation of team reflection sessions. They serve as an effective implementation strategy by promoting the implementation of the intervention (Pettersen et al., 2024).

### 3.7 Data nurse intervention support

At the team level, data champions will serve as local champions to facilitate adoption within their teams. Team leaders will coordinate team members' and older adults' participation throughout the study. At the organizational level, informative meetings were held prior to the study to engage participating organizations. Throughout the study, researchers will provide ongoing support to teams through regular monthly contact with team leaders and data champions.

### 3.8 Participants and eligibility criteria

District nursing teams members are eligible to participate if they are actively involved in patient intake, goal-setting, and care evaluation processes. Within teams allocated to the intervention group, all team members used the shared decision-making tools and dashboard as part of usual team practice. Individual team members could, however, decide whether or not to participate in the study's data collection (e.g., questionnaires and interviews). Older adults receiving care from team members who did not take part in data collection remained eligible for inclusion, as they still received the intervention through their team. Older adults are eligible if they are aged 65 years or older and currently receive district nursing care. Older adults must have sufficient cognitive capacity to provide informed consent and participate in interviews and in-person-centered shared decision-making. In addition, older adults must be able to complete the patient preparatory shared decision-making tool in Dutch, with help from their informal caregiver, if needed (Pel-Littel et al., 2020). Older adults are excluded if they have a life expectancy of less than three months. Also, older adults with a life expectancy of <3 months or with severe physical or cognitive impairments will be excluded from the study.

### 3.9 Allocation of participants

District nursing teams from participating organizations could voluntarily sign up for the study. Allocation to the intervention or control group was determined by the participating organizations, based on pragmatic factors such as team availability, staffing, and time resources. This pragmatic approach, which includes both an intervention and a control group, allows for assessing feasibility and exploring preliminary signals of effectiveness. It aligns with Eldridge et al., who highlight the importance of early impact indicators in feasibility studies of complex interventions (Eldridge et al., 2016). This design enables and informs the refinement of the intervention and future study planning.

### 3.10 Intervention group procedures

Teams assigned to the intervention group participate in the full Data Nurse intervention. Upon enrolment, team members will complete the e-learning module on person-centered shared decision-making and designated data champions will attend the train-the-trainer workshop. Teams then execute the intervention during five months, in which they will integrate the shared decision-making approach and dashboard into their routine care practice. Team leaders will register participating older adults in an online data registry, Castor, for data collection. Castor is a cloud-based platform that facilitates data collection, management, and analysis for clinical research and healthcare (CastorElectronicData Capture, 2025). Older adults in the intervention group will receive the patient preparatory tool shared decision-making prior to their initial care conversations. Throughout the intervention period, data champions will facilitate monthly team reflection sessions using dashboard insights.

### 3.11 Control group procedures

Teams assigned to the control group will provide care as usual. They will not receive the e-learning program, the patient preparatory tool shared decision-making, or training on or access to the Data Nurse dashboard. Team leaders will coordinate research activities and register older adults in Castor. Control teams will participate only in data collection.

### 3.12 Ethical approval and informed consent

Ethical approval for this study was granted by the Medical Ethics Committee of Amsterdam UMC [2024.0956]. All research procedures involving human participants will be conducted in accordance with institutional guidelines and the principles of the Declaration of Helsinki (World Medical Association, 2013). Written informed consent will be obtained from all participants prior to data collection. Participation is entirely voluntary, and individuals may withdraw from the study at any time without consequences for their care or professional role. Data collection up to the point of withdrawal will be retained unless the participant explicitly requests its removal. Prior to providing consent, all potential participants will receive written study information in plain language. For district nursing team members, this includes an information letter describing the study purpose, procedures, time investment, and data use. For older adults, study information will be provided in plain language, with the opportunity to ask questions and, where needed, to involve an informal caregiver in the decision to participate.

### 3.13 Outcomes

Outcomes will be examined at the district nursing team, team member, and older adult levels for both intervention and control groups. All effectiveness outcomes are exploratory and intended to inform a future randomized controlled trial: no formal hypothesis testing will be conducted on effectiveness measures.

### 3.14 Primary outcomes

Primary outcomes concern feasibility, acceptability, fidelity, barriers and facilitators, and experiences related to the Data Nurse intervention. These outcomes will be assessed using a mixed-methods approach combining quantitative instruments and qualitative data collection.

**Feasibility** refers to the extent to which the intervention can be successfully used or carried out by the intended users in routine district nursing practice. Feasibility will be measured in district nursing team members and older adults from the intervention group using the Feasibility of Intervention Measure (FIM), a five-item Likert scale assessing perceived implement ability (Weiner et al., 2017). The feasibility of the two components of the total intervention will be assessed in team members; the feasibility of the patient preparatory tool shared decision-making will be assessed in older adults.

**Acceptability** concerns the degree to which nursing professionals and older adults consider the intervention appropriate, relevant, and worthwhile (Sekhon et al., 2017). Acceptability will be measured in nursing professionals and older adults from the intervention group using a questionnaire based on the Theoretical Framework of Acceptability (TFA), assessing affective attitude, perceived burden, ethicality, coherence, perceived effectiveness, self-efficacy, and opportunity costs on a 5-point Likert scale (Sekhon et al., 2017). The acceptability of the total intervention will be assessed in nursing professionals; the acceptability of the patient preparatory tool shared decision-making will be assessed in older adults.

**Fidelity** addresses whether the intervention is delivered as intended, including completion of training, use of the shared decision-making approach and patient preparatory tool shared decision-making, and dashboard engagement. Fidelity will be measured in nursing professionals from the intervention group using self-developed questionnaires (Supplementary File 3), tracking adherence indicators such as e-learning completion, use of stepwise person-centered shared decision-making from the e-learning, patient preparatory tool shared decision-making usage, and dashboard interactions. Teams complete these questionnaires on Castor one week before monthly online fidelity meetings with researchers, data champions, and team leaders. During these meetings, participation and delivery will be discussed using the Kotter framework (Kotter, 1996). Team logbooks will be used to document participation and implementation challenges throughout the intervention period, complementing the structured meeting discussions. Fidelity is considered high when ≥80% of teams completed the planned activities, consistent with commonly used thresholds for high intervention fidelity in implementation research (Borrelli, 2011).

**Barriers and facilitators** are factors that hinder or support the implementation of an intervention, encompassing motivation, capability, organizational readiness, and context (Huijg et al., 2014). Barriers and facilitators will be identified among nursing professionals in the intervention group using the Theoretical Domains Framework (TDF) questionnaire, covering these domains on 5-point Likert scales (Huijg et al., 2014).

**Experiences** will be measured regarding how nursing professionals and older adults perceive and reflect on working with the intervention in daily practice, including how it fits within existing workflows, how it shapes conversations and decision-making with older adults, and what unexpected effects, learning points or adaptations emerge during use. This compliments the other primary outcomes by providing in-depth, contextualized insights that cannot be caputered through standardized scales such as FIM, TFA en TDF.

A self-developed questionnaire captures nursing professionals' experiences with using the stepwise person-centered shared decision-making method in practice (supplementary file 4.).

Qualitative data on all primary outcomes will be collected through focus groups with nursing professionals from the intervention teams and individual interviews with older adults from the intervention group, exploring perceived feasibility, acceptability, barriers and facilitators, and experiences with the intervention including the patient preparatory tool shared decision-making and the dashboard. Focus groups and interviews will be conducted halfway through the study.

### 3.15 Secondary outcomes

Secondary outcomes are the preliminary effectiveness of the Data Nurse intervention, assessed in both intervention and control groups.

Self-reported independent functioning will be measured using the selected domains of the TOPICS-SF that assess independent functioning (Santoso et al., 2018), a patient-reported outcome measure assessing health and functioning across multiple domains, including physical health, mental health, social support, and overall wellbeing. In the intervention group, this is measured through the patient preparatory tool shared decision-making, which incorporates the TOPICS-SF items. In the control group, older adults will complete the selected domains of the TOPICS-SF separately. As the instrument is administered through different channels, group comparability will be considered in the analysis.

Patient-reported shared decision-making reflects the level of shared decision-making in the clinical encounter from the older adults' perspective. This will be measured using the CollaboRATE questionnaire (Elwyn et al., 2013), a three-item instrument, scored from 0 to 9, that evaluates the extent to which older adults perceive their concerns to be heard and integrated into care decision-making.

District nursing care delivery patterns will be extracted from electronic nursing record data for both groups, including visit frequency, care delivery mode (in-person, phone, video), use of healthcare technology, and total care hours. Additionally, three data-informed indicators are extracted from the electronic nursing record system to explore the extent to which care activities are aligned with promoting patient independence: (1) the proportion of nursing interventions aimed at promoting independent functioning, as documented in the care plans; (2) the proportion of older adults showing improvement in care need scores between intake assessment and evaluation, or between successive evaluations; and (3) the proportion of older adults who achieved their individually set target scores per care need during the study period.

### 3.16 Harms

The Data Nurse intervention is a non-pharmacological, educational and behavioral intervention embedded in routine district nursing care practice. Given its nature, no physical or medical harm to participants is anticipated. However, potential unintended effects will be monitored throughout the study. For older adults, the shared decision-making process may surface unmet care needs or emotional concerns related to declining independence, which could cause temporary distress. For district nursing team members, increased demands on time and workload associated with the intervention activities (completing the e-learning, facilitating shared decision-making conversations, and participating in team reflection sessions) may contribute to perceived burden. These potential harms will be explored through qualitative data collection (interviews and focus groups) and discussed during the monthly fidelity meetings with team leaders and data champions. No formal adverse event reporting procedures or data monitoring committee has been established, as this is a low-risk feasibility study of a behavioral intervention. Participants may withdraw from the study at any time without consequences for their care or professional role.

### 3.17 Sample size

As this is a feasibility study, the sample size is not based on a formal power calculation. However, it is justified by pragmatic considerations and the available study population, in line with recommendations for feasibility studies of complex interventions (Eldridge et al., 2016). The study involves four organizations, each contributing 14 district nursing teams: 8 in the intervention group and 6 in the control group. Up to 168 district nursing team members are eligible, based on an average of 12 professionals per team. A participation rate of 50–65% is expected among district nurses (L'Ecuyer et al., 2023), resulting in approximately 84–109 participants. Each team supports an average caseload of 30 older adults (aged ≥65), yielding approximately 420 eligible participants across all teams. Based on expected recruitment rates in feasibility studies, a participation rate of approximately 50–70% is anticipated (Eldridge et al., 2016).

### 3.18 Recruitment and participation

Recruitment will follow a multi-level approach. District nursing care organizations affiliated with the Data Nurse project will be invited to participate. Within these organizations, contact persons will distribute invitations to district nursing teams through internal communication channels such as newsletters, online bulletin boards and team meetings. Interested teams will be contacted by the research team, who will provide detailed information about the study and participation requirements. Individual team members within participating teams will decide voluntarily whether to participate in the research activities.

**Fig. 3:**
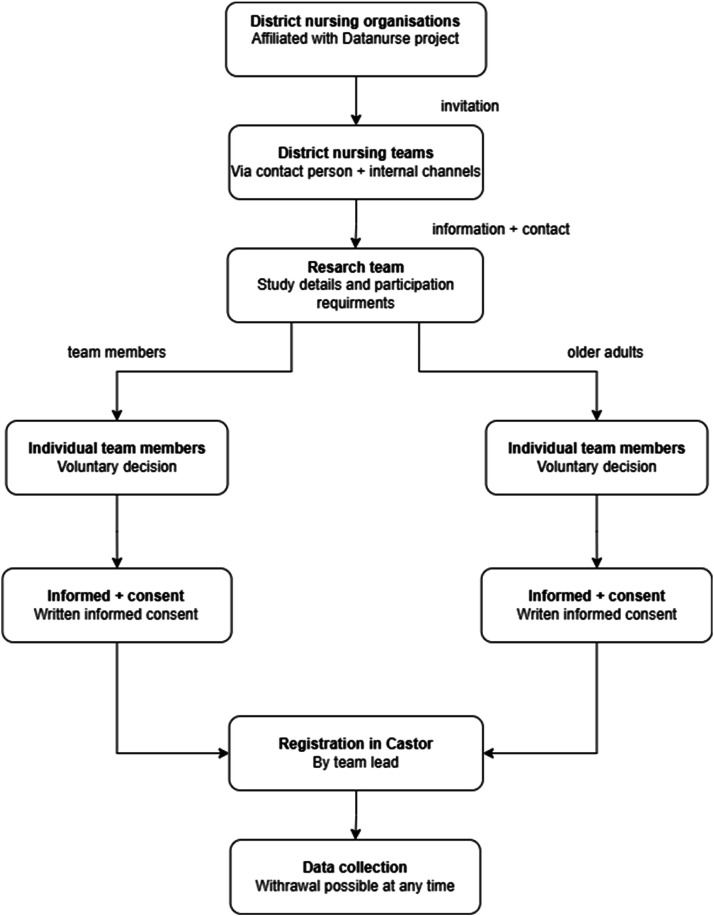
Recruitment flowchart.

Older adults will be recruited by team members from participating teams, who will identify eligible older adults from their caseload and provide them with study information and an information letter. The recruitment procedure is visualized in Fig. 3.

Upon agreement to participate, team members and older adults will receive an information letter and provide written informed consent. Team leads register participating older adults in Castor for data collection. Participants can withdraw from the study at any time without consequences for their care.

#### 3.18.1 Data collection

Data will be collected over a five-month period from June to November 2025, using a combination of questionnaires, interviews, focus groups, and electronic nursing record data. Table 1. presents the complete schedule of enrolment, interventions, and measurements. Team members will complete questionnaires online through Castor. Older adults will complete paper-based questionnaires, which will be entered into Castor by researchers. Primary outcomes (feasibility, acceptability, fidelity, barriers and facilitators, and experiences) will be assessed exclusively in the intervention group, as these relate directly to the use of the intervention. Secondary outcomes (self-reported independent functioning, patient-reported shared decision-making, and nursing care delivery patterns) will be assessed in both groups to allow comparison.

**Table 1:** Schedule of enrolment, interventions, and assessments (SPIRIT).

		Population	Instrument	Trial period						
				Enrolment	Allocation	Post-allocation				Close-out
Timepoint				-t₁	0	*t*₁	*t*₂	*t*₃	*t*₄	*t*₅
				*May*	*Jun*	*Jul*	*Aug*	*Sep*	*Oct*	*Nov*
**Enrolment**										
	Eligibility screening	DNT members		**X**						
	Informed consent	DNT members + older adults		**X**						
	Baseline data collection	DNT members			**X**					
	Baseline data collection	Older adults			**X**					
	Allocation	DNT			**X**					
**Intervention preparation**										
	Use patient preparatory tool shared decision-making	Older adults (I)			**X**					
	shared decision-making e-learning	DNT members (I)			**X**					
	Follow dashboard workshop	Data champions + older adults (I)			**X**					
**Intervention or comparator**										
	Data Nurse intervention cycle*	DNT member (I)			**X**	→	→	→	→	
	Care as usual	DNT members (C)			**X**	→	→	→	→	
**Assessments: Primary outcomes**										
***Feasibility***										
	Feasibility of intervention	DNT members (I)	*FIM*		**X**					**X**
	Feasibility of patient preparatory tool shared decision-making	Older adults (I)	*FIM*		**X**					**X**
***Acceptability***										
	Acceptability of intervention	DNT members (I)	*TFA*		**X**					**X**
	Acceptability of patient preparatory tool shared decision-making	Older adults intervention team	*TFA*							**X**
***Fidelity***										
	Adherence to intervention components	DNT members (I)	*Self-developed questionnaire*			**X**	**X**	**X**	**X**	**X**
	Delivery monitoring (Kotter framework)	Data Champions (I)	*Monthly meetings*			**X**	**X**	**X**	**X**	**X**
***Barriers and facilitators***										
	Implementation factors	DNT members (I)	*TDF*		**X**					**X**
	shared decision-making -specific experiences and barriers	DNT members (I)	*Self-developed*							**X**
***Experiences***										
	Experiences with shared decision-making	DNT members (I)	*Self-developed questionnaire*							**X**
	Experiences with shared decision-making	Older adults (I)	*Interviews*							**X**
	Intervention experiences	DNT members (I)	*Focus groups*							**X**
**Assessments: Secondary outcomes**										
Self-reported independent functioning	Health and functioning across domains	Older adults (I+C)	*Modified TOPICS-SF*		**X**					**X**
Patient-reported shared decision-making	Level of shared decision-making from patient perspective	Older adults (I+C)	*CollaboRATE*		**X**					**X**
Nursing care delivery patterns	Visit frequency, delivery mode, care hours	DNT members (I+C)	*ENR data*		**X**					**X**
**Additional data collection**										
**Care as usual**	Experiences with usual care	DNT members (C)	*Interviews*							**X**

I=Intervention Group; C=Control Group. DNT: District Nursing Team. SDM: Shared Decision-making.

##### 3.18.1.1 District nursing team members

At baseline, characteristics of participants in nursing teams will be collected. For team members, these include year of birth, gender, position, years of experience, contract type, and organization. Additional qualitative information will be collected on team members' educational backgrounds.

Team members in the intervention group will complete FIM questionnaire on feasibility (Weiner et al., 2017), TFA on acceptability (Sekhon et al., 2017), barriers and facilitators (Huijg et al., 2014), fidelity and experiences with the shared decision-making method (self-developed questionnaires; Supplementary Files 3. and 4,), at the time points specified in Table 1. At the end of the intervention period, team members will participate in focus groups to explore their experiences with the intervention, including perceived feasibility, acceptability, and barriers and facilitators.

Team members in the control group will participate in interviews about care as usual at the end of the study period. District nursing care delivery patterns, including visit frequency, care delivery mode, and total care hours, will be extracted from the electronic nursing record for both groups at baseline and at the end of the study period.

##### 3.18.1.2 Older adults

At baseline, characteristics of older adults who participate will be collected. These include year of birth, gender, marital status, living situation, highest level of education completed, country of birth, reason for receiving care, frequency of contact with district nursing, financial concerns, and care or support from the municipality. Additional qualitative information will be collected on older adults' living situation and municipal support.

Older adults in the intervention group will complete the patient-shared decision-making preparatory tool, based on the TOPICS-SF and covering self-reported independent functioning, general health, pain, emotional well-being, social activities, quality of life, and personal goals and wishes. In addition, they will complete the TFA questionnaire on acceptability of the patient preparatory tool shared decision-making and the CollaboRATE questionnaire on patient-reported involvement in shared decision-making. Individual interviews will be conducted to explore their experiences with person-centered shared decision-making.

Older adults in the control group will complete selected domains of the TOPICS-SF, specifically the self-reliance domain (10 items) and the social activities domain (1 item, to measure self-reported independent functioning, as well as the CollaboRATE questionnaire.

## 4 Data analysis

Quantitative and qualitative data will be analyzed separately and subsequently integrated to provide a comprehensive understanding of the intervention's feasibility, acceptability, fidelity, experiences, barriers, and facilitators, and preliminary effectiveness.

### 4.1 Qualitative analysis

Interview and focus group data will be analyzed using thematic analysis following the six-phase framework of Braun and Clark (Braun and Clarke, 2014). Audio recordings will be transcribed verbatim using Microsoft Teams and imported into MAXQDA (MAXQDA, 2024) for systematic coding and theme development. An inductive coding approach will be used to identify patterns related to the outcomes to Data Nurse intervention. To enhance analytic rigor, two researchers will independently code the transcripts, with discrepancies resolved through discussion. Data triangulation between patient interviews and nursing professionals' focus groups will strengthen the comprehensiveness.

### 4.2 Quantitative analysis

Paper questionnaires will be manually entered into Castor to ensure a complete central database. Data will be checked for outliers, inconsistencies, and missing values prior to analysis. Likert scale responses will be treated as ordinal variables. All statistical analyses were conducted using R, version 4.4.3, an open-source software environment for statistical computing and graphics (Posit team, 2025). Demographic characteristics of participants will be summarized using descriptive statistics (means, standard deviations, frequencies, and percentages). Primary outcomes, including feasibility (FIM), acceptability (TFA), fidelity, and barriers and facilitators (TDF), will be analyzed descriptively. Retention and participation rates will be calculated as percentages of initial enrolment to assess engagement throughout the study. For secondary outcomes (self-reported independent functioning, patient-reported shared decision-making, and nursing care delivery patterns), changes between baseline and follow-up will be explored descriptively. Effect sizes and confidence intervals will be reported to inform the design of a future effectiveness trial.

### 4.3 Integration

Quantitative and qualitative findings will be integrated using a narrative synthesis approach. (Fetters et al., 2013)Data triangulation between older adult interviews, team member focus groups, and quantitative outcomes will be conducted to provide a comprehensive understanding of the intervention's feasibility and preliminary effectiveness (Creswell, 2022).

### 4.4 Data management

All study data will be stored on the secure research drive of Amsterdam University Medical Center, in compliance with institutional data protection policies. Questionnaire data will be collected and managed through Castor. Paper questionnaires completed by older adults will be manually entered into Castor by researchers. Transcripts from interviews and focus groups will be coded and stored on the secure research drive, separately from participant identifiers. All data will be coded using a subject ID; the key linking subject IDs to participant identities will be stored separately and accessible only to authorized researchers. Data will be retained for a minimum of 10 years in accordance with institutional and regulatory guidelines. Secure data transfers will be managed via SURF FileSender.

## 5 Discussion

This protocol presents the feasibility study of the Data Nurse intervention. This novel complex intervention combines person-centered shared decision-making with data-informed care to support independent functioning among older adults receiving district nursing care. To our knowledge, no intervention combining these two components has been previously developed or evaluated in this setting; however, they have been shown to be effective in other clinical settings.

The study has several strengths. The real-world implementation across multiple organizations increases the relevance of the findings for everyday district nursing practice. The mixed-methods approach combines validated instruments with in-depth qualitative exploration, allowing for comprehensive assessment of both process and preliminary outcomes. The involvement of data champions as local champions reflects implementation science principles for sustainable practice change (Pettersen et al., 2024), and responds to identified needs for organizational support when introducing data-informed practices in district nursing care (Veldhuizen et al., 2024a). The dual stakeholder perspective, assessing outcomes from both team members and older adults, recognizes that feasibility depends on the experiences of all involved. Finally, the study prioritizes contextual factors and participant experiences over formal hypothesis testing, in line with Medical Research Council guidance for evaluating complex interventions in their early stages (Skivington et al., 2024).

### 5.1 Limitations and challenges

Several limitations should be acknowledged. The non-randomized design may introduce selection bias, though this reflects the operational constraints of district nursing practice and is consistent with recommendations for feasibility studies (Eldridge et al., 2016). Self-reported fidelity measures may be subject to social desirability bias, which will be mitigated through triangulation with team logbooks and structured monthly meetings. The five-month intervention period may be insufficient to capture the full adoption of a complex behavioral intervention. The comparability of the selected domains of the TOPICS-SF data between groups warrants attention, as the instrument is administered through different channels. Finally, the limited number of clusters may constrain the statistical analysis of secondary outcomes. The findings will inform whether the intervention warrants a larger effectiveness trial, which components require refinement, and what implementation strategies are needed across district nursing contexts.

## 6 Conclusions

This feasibility study will provide essential evidence on the feasibility and preliminary results of the Data Nurse intervention, a novel complex intervention combining person-centered shared decision-making with data-informed care to support independent functioning among older adults receiving district nursing care. Findings will inform intervention refinement.

## Funding

This work is supported by 10.13039/501100001826ZonMw (Grant Number 10040022010003). The funding agency had no role in the study design, methods, and subject requirements; in the collection, analysis, and interpretation of data; in the writing of the report; or in the decision to submit the article for publication.

## Data statement

The datasets generated and/or analysed during this study will not be publicly available due to the sensitive nature of the data and the need to protect participant confidentiality. Data will be available upon reasonable request, following publication of the study results, subject to ethical approval and the signing of a Data Sharing Agreement (DSA). Requests for data access should be directed to the corresponding author. Data will be retained for a minimum of 10 years in accordance with institutional guidelines of Amsterdam UMC.

## Study status

Study initiation commenced on June 2025. Recruitment and inclusion of district nursing teams took place in June 2025. Patient recruitment and inclusion will occur between June 15th and the end of September 2025. Qualitative analysis of interview and focus group data will be conducted from November to April 2026. Quantitative data analysis, including patient-reported outcome measures (PROMs) and care metrics, will be carried out between November 2025 and April 2026.

## Declaration of generative AI use

During the preparation of this work, the author(s) used Claude (Anthropic) to assist with structuring and refining the manuscript text and improving language and readability. All study design decisions, scientific content, data collection procedures, and analytical approaches were determined entirely by the authors. After using this tool, the author(s) reviewed and edited all content as needed and take full responsibility for the content of the published article.

## CRediT authorship contribution statement

**Sigrid Wulfse-Huisman:** Writing – review & editing, Writing – original draft, Project administration, Methodology, Investigation, Formal analysis, Data curation, Conceptualization. **Xenia Yocarini:** Writing – review & editing, Writing – original draft, Methodology, Investigation, Formal analysis, Data curation, Conceptualization. **Jessica Veldhuizen:** Writing – review & editing, Supervision, Methodology, Conceptualization. **Koen van den Braak:** Writing – review & editing, Project administration, Methodology, Investigation, Data curation, Conceptualization. **Mariëlle Zondervan-Zwijnenburg:** Writing – review & editing, Methodology, Formal analysis. **Ruth Pel-Littel:** Writing – review & editing, Supervision, Funding acquisition, Conceptualization. **Bianca Buurman:** Writing – review & editing, Supervision, Funding acquisition, Conceptualization. **Nienke Bleijenberg:** Writing – review & editing, Supervision, Methodology, Funding acquisition, Conceptualization.

## Declaration of competing interest

The authors declare that they have no known competing financial interests or personal relationships that could have appeared to influence the work reported in this paper.

## Appendix Supplementary materials

Image, application 1

Image, application 2

Image, application 3

Image, application 4

Image, application 5

Image, application 6

Image, application 7

Image, application 8

Image, application 9

Image, application 10
