# Supplementary material for: Person-centered shared decision-making and data-informed district nursing care to enhance independence: Protocol for a feasibility study
Source: Int J Nurs Stud Adv. 2026 Jun 1;11:100569. doi: 10.1016/j.ijnsa.2026.100569 (PMC13266195; doi:10.1016/j.ijnsa.2026.100569)
Supplement: Supplementary file 1 [file mmc1.docx]

**Checklist for developing and evaluating complex interventions**

This checklist is intended as a tool to help researchers prepare funding applications, research protocols and journal publications. It may also help reviewers to assess whether the recommendations have been followed.

| **Item** | **If NO, please justify**  **If YES, Briefly describe how this has been addressed** | **Reported on page number(s)** |
| --- | --- | --- |
| **Addressing uncertainties**   1. Have you determined the aim(s)/purpose(s) of the intervention? 2. Have you identified the key uncertainties given existing evidence about the intervention and the context in which it will be tested or implemented? 3. Do the research questions and methods address the key uncertainties? 4. Does the choice of research perspective (efficacy, effectiveness, theory-based, systems) reflect the key uncertainties that have been identified? | Yes, the aim of the data nurse intervention is to improve patient support data-informed shared decision-making in district nursing via a dashboard on patient independence. Key uncertainties regarding acceptability, feasibility, and preliminary effectiveness have been identified from literature and formative work. Research questions and mixed-methods design directly address these uncertainties. A feasibility perspective was chosen, reflecting the early-phase evidence base and the need to inform intervention refinement | 5-8 |
| **Engaging stakeholders**   1. Have you engaged stakeholders in the design/identification of the intervention and the development of the research protocol? 2. Have you engaged stakeholders in the conduct of the research and the dissemination of findings? 3. Have all stakeholders declared any potential conflicts of interest? | Yes, district nursing teams, data champions and patients were involved in co-designing components of the data nurse intervention (dashboard, e-learning and patient shared decision-making preparatory tool). Stakeholders will also be involved in the conduct of the study. all stakeholders have declared any potential conflicts of interest | 13-17 |
| **Considering context**   1. Have you identified all the dimensions of context that may influence how the intervention achieves its effects? 2. Have you considered how context may affect the scaling up of scaling out of the intervention? | Yes, contextual dimensions including organisational culture, digital infrastructure, team composition, patient complexity and regulatory frameworks in Dutch district nursing have been identified and described. Contextual factors relevant to potential scale-up have been considered in this feasibility design (e.g. variation across organisations, urban vs rural settings) | 8-12 |
| **Developing and refining programme theory**   1. Have you developed a programme theory for your intervention that describes the key components and mechanisms of the intervention and how it interacts with the context in which it will be implemented? 2. Have you updated the programme theory to incorporate the new evidence gathered by the study? | Yes, a programme theory describing the key components and mechanisms of the DataNurse intervention has been developed. The programme theory will be updated based on evidence gathered during this feasibility study. | 13-15 |
| **Refining the intervention**   1. Have you refined the intervention so that it is optimised for the context in which it will be implemented? 2. Have you specified how far and in what ways the intervention can be refined during implementation without undermining the programme theory? | Yes, the intervention was iteratively refined through formative work. The protocol specifies which elements of the intervention are fixed (core components) and which may be adapted (e.g. timing of team meetings, dashboard visualisations) to optimise fit with local context without undermining the programme theory | 9-12 |
| **Economic considerations**   1. Have you considered whether the value of the evidence, in terms of informing future decision-making, justifies the cost of the research? 2. Have you identified an economic evaluation framework that is appropriate to the expected outcomes of the intervention? | Yes, the value of this feasibility evidence for informing the decision to proceed to a larger trail has been considered and deemed to be justify the research cost. An economic evaluation framework is not formally included at this feasibility stage, but resource use data will be collected to inform economic analysis in a definitive trial | 17-25 |
| **Phase-specific considerations**  **Developing interventions:** Have you used a formal framework (such as INDEX) to guide development of the intervention?  **Identifying interventions**: For policy and practice interventions, have you performed an evaluability assessment to determine whether and how an evaluation should be undertaken?  **Feasibility:** Have you defined and used clear progression criteria to guide decisions about whether to proceed to an evaluation study?  **Evaluation:** Have you chosen an appropriate study design to answer the research questions and provide robust evidence to inform decision-making about further intervention refinement, evaluation or implementation?  **Implementation**: Have constraints and enablers of implementation been considered at all phases, from intervention development, through feasibility and effectiveness testing, to large scale roll-out? | Developing interventions: YES. The Data Nurse intervention was developed through systematic phases of exploration, co-design, and refinement (pp. 9-10). Although the INDEX framework was not explicitly applied, the development process followed comparable systematic principles aligned with MRC guidance  Feasibility: PARTIAL. A fidelity threshold of ≥80% has been pre-specified as a progression criterion (p. 15). Full progression criteria for a future definitive trial (including recruitment rate, retention, and outcome measure completion) have not yet been formally defined, as one aim of this feasibility study is to generate the empirical data needed to establish appropriate thresholds. Criteria will be developed with stakeholders following study completion.  Evaluation: YES. A pragmatic non-randomized multicenter feasibility design was chosen as appropriate to the research questions and early-phase evidence base (p. 7).  Implementation: YES. Constraints and enablers of implementation have been considered throughout, including training requirements, data champion roles, and organisational variation (pp. 12-14). | 8-15 |

Permission acknowledgement: Copyright © 2021 Skivington et al. This work was produced by Skivington et al. under the terms of a commissioning contract issued by the Secretary of State for Health and Social Care. This is an Open Access publication distributed under the terms of the Creative Commons Attribution CC BY 4.0 licence, which permits unrestricted use, distribution, reproduction and adaption in any medium and for any purpose provided that it is properly attributed. See: <https://creativecommons.org/licenses/by/4.0/>.
