# Supplementary material for: Person-centered shared decision-making and data-informed district nursing care to enhance independence: Protocol for a feasibility study
Source: Int J Nurs Stud Adv. 2026 Jun 1;11:100569. doi: 10.1016/j.ijnsa.2026.100569 (PMC13266195; doi:10.1016/j.ijnsa.2026.100569)
Supplement: Supplementary file 2 [file mmc2.docx]

**Shared Decision-Making Preparatory Tool**

What is important to you?

Please bring the completed questionnaire with you to your appointment with your healthcare provider

By completing this questionnaire, you will be better prepared for a conversation with your healthcare provider. This allows you to discuss what is important to you and decide together what suits you best. Your healthcare provider has professional expertise, but you know your own situation best

As you age, a lot may come your way. You might experience various discomforts and/or illnesses that can require significant effort from both you and your surroundings. To find appropriate solutions, including possible support, care, and/or treatment, it is important for your healthcare provider to be well informed about your situation.

**Your own goals and wishes**
What discomforts and complaints do you have? What is important to you? What are your wishes? Share these with your healthcare provider because you know your situation best. How you think about your own situation might not be the same as how your healthcare provider views it. You may prioritize different things. For example, do you want to be able to go out more or experience less pain? Discuss this with your healthcare provider.

**Discussing what suits you best**

You can then, based on your personal situation and the medical or care knowledge of your healthcare provider, discuss the possible solutions for you. You can also talk about the pros and cons of those solutions in relation to your life. This will help both of you make a joint decision on the options that best suit you.

**INSTRUCTIONS FOR FILLING OUT THE PATIENT SDM PREPARATORY TOOL**

The questions on the following pages will help you and your healthcare provider gain insight into your personal situation and what you find important to discuss.

- The tool consists of two parts. We kindly ask you to fill out part 1 before your conversation. Part 2 can be completed during the conversation.
- Filling out the tool will take you between 10 and 30 minutes.
- If you find it difficult to understand or complete the questions, ask for help from your partner, a family member, or a friend. However, it is your personal experience of your situation that matters.
- Read each question carefully before choosing an answer.
- Once you are done, check if you have missed any questions.

*Your healthcare provider can be your doctor, district nurse, practice assistant, or another healthcare professional.*

**PART ONE**

**Name:**
**Date of Birth:**
**Today's Date:**

Choose the answer that best fits your situation.

**1. General Health**

- On a scale of 0–10: how is your general health?
  (0 = completely sick, 10 = completely healthy)

1 2 3 4 5 6 7 8 9 10
(circle the number that best applies)

- What would need to change for your health to go up by one point?

**2. Pain/Discomfort**

- How do you currently experience pain or discomfort?
  (None, A little, Moderate, Severe, Extreme)

□ □ □ □ □

**3. Memory**

- Do you have any concerns about your memory?
  □ Yes □ No

**4. Daily Life**

- Can you do the following independently?

| **Activity** | **Yes, without any difficulty** | **Yes, with some difficulty** | **Yes, with much difficulty** | **No, only with help from others** |
| --- | --- | --- | --- | --- |
| Dressing and undressing | □ | □ | □ | □ |
| Getting up from a chair | □ | □ | □ | □ |
| Washing and drying your whole body | □ | □ | □ | □ |
| Climbing and descending stairs | □ | □ | □ | □ |
| Walking outside (with a cane or walker if needed) | □ | □ | □ | □ |
| Taking care of your feet and toenails | □ | □ | □ | □ |
| Doing light household tasks (e.g., dusting, tidying up) | □ | □ | □ | □ |
| Doing grocery shopping | □ | □ | □ | □ |
| Taking your medication | □ | □ | □ | □ |
| Using public or personal transportation | □ | □ | □ | □ |

**5. How I Feel**

- How often, over the past 4 weeks, did I feel…
  (Always, Often, Sometimes, Rarely, Never)

| **Feeling** | **Always** | **Often** | **Sometimes** | **Rarely** | **Never** |
| --- | --- | --- | --- | --- | --- |
| Happy? | □ | □ | □ | □ | □ |
| Calm and relaxed? | □ | □ | □ | □ | □ |
| Very nervous? | □ | □ | □ | □ | □ |
| So down that nothing could cheer me up? | □ | □ | □ | □ | □ |
| Lonely? | □ | □ | □ | □ | □ |

**6. Social Activities**

- How often have your physical health or emotional problems, over the past 4 weeks, hindered your social activities (such as visiting friends or close family members)?
  (Always, Often, Sometimes, Rarely, Never)

□ □ □ □ □ □

**7. The Conversation and My Wishes and Expectations**

- Which of the following topics do you find important to discuss with your healthcare provider?
  Examples:
  - The future
  - Medication
  - New or worsening symptoms
  - Pain/Discomfort
  - Memory
  - Daily life
  - How I feel
  - Social activities
  - Staying independent
  - Resuscitation (whether or not to perform)
  - End of life
- What else would you like to discuss?

**8. What Is Important to Me in Life**

The following questions are about what is important to you. If we know what matters to you, we can better tailor your care to your situation.

What makes me happy:

What I hope for:

What I fear:

What I would like to continue doing:

What I want to (be able to) do again:

What I would like to experience less discomfort from:

If an important decision needs to be made about my health, do I prefer to make that decision together with my loved one(s)?

If yes; with who?

The next question is about your ‘quality of life.’ This refers to how you feel about your life, whether you are satisfied with your life, whether you enjoy your life, and whether your life brings you fulfillment.

- On a scale of 1 to 10: how satisfied am I with my life?
  (1 = completely dissatisfied, 10 = completely satisfied)

1 2 3 4 5 6 7 8 9 10
(circle the number that best applies)

- What would need to change for your satisfaction to go up by one point?

**9. Closing Questions**

- Did anyone help you fill out this questionnaire?
  □ No, I filled it out myself.
  □ Yes, someone helped me fill it out:
  - Partner
  - Daughter/Son
  - Friend
  - Other:
- If yes, what kind of help did you receive?
  □ Someone else wrote the answers down.
  □ I chose the answers myself.
  □ I chose the answers with someone else and they wrote them down.
  □ Someone chose and wrote down the answers for me.

It is important that you bring the completed questionnaire to your appointment with the healthcare provider. Thank you in advance!

**Do you have an informal caregiver?**

Below is a question for informal caregivers

For caregivers

The healthcare provider is also interested in how things are going with people who care for their loved ones (partner, parent, family member, friend). The support you provide is important, and you must be able to sustain it.
The healthcare provider will also ask how your own health is. We think about both physical and mental health and how you are feeling.

- As a caregiver I feel …………….
- Are there things you are concerned about regarding your loved one?
- What do you hope for your loved one?

**PART TWO**

The following section can be filled out during the conversation with the healthcare provider.

**Which goals do we consider most important for support, care, and/or treatment?**

See for example the answers at item 8.

For each goal: What options are available? What can I do myself?

Which (digital) tools can help? What can my environment do? What can the healthcare provider do?

For each option: What are the advantages and disadvantages of the different possibilities? What is the impact on my daily life?

What we decide together is the best fit for my situation and preferences:

**Steps for SDM**

**Step 1: Preparation**

Introduce yourself to your client. Explain what community nursing can offer. For example, tell your client that:

- Community nursing is focused on care and well-being.
- Community nursing discusses not only health issues but also self-sufficiency.
- Client goals are an important starting point for care.
- Together, you will discuss what care is needed.
- The focus is on promoting self-sufficiency in the formulation and execution of care.
- With the tool, the client can outline their health goals, such as quality of life and self-sufficiency goals.

Ask your client to fill out the tool. The client fills it out at home. This helps the client prepare and provides insight into:

- Perceived health.
- Topics to discuss.
- Goals and values.
- The possibilities of the caregiver.

You also prepare yourself. For example, you may have relevant information from a nursing handover or a medical record. Read the Preparatory Tool carefully.

**Step 2: Life Goals and Values**

- Specify which topics/health issues will be discussed and mention that before proceeding, you would like to first discuss what quality of life means for your patient.
- Which life goals and values are important?
- Discuss what the patient has written in the tool.

**Helpful Phrases:**

- "When you tell me more about what’s important to you, we can adjust the care better to suit you."
- "Can you tell me what matters most to you?"
- "What do you hope for?"
- "What are you afraid of?"
- "Do you prefer someone to support you in making decisions? If so, who would you like to involve?"

**Step 3: Choosing Treatment, Care, or Well-being Goals**

- Summarize what’s most important for your client and which health issues are involved.
- Discuss what you will work on together.
- Formulate care or well-being goals with the client.

**Helpful Phrases:**

- "Let me summarize what we have discussed so far."
- "Can you tell me in your own words what you would like to achieve?"
- "What is the most important thing for you right now?"

**Step 4: Discussing Options**

- Review the options to achieve the goal.
- Consider not only care options but also well-being options.
- Doing nothing can also be an option.
- Ask the patient and possibly the caregiver what options they see or could possibly take on themselves.
- Discuss the pros and cons of each option.
- Discuss the impact of each option on daily life.
- Discuss which other healthcare professionals could be involved and what they can contribute.
- Work together with your client to explore their preferences.

**Helpful Phrases:**

- "Based on our conversation, these are the possible options..."
- "In your situation, these would be the options that fit..."

**Step 5: Decision-Making**

Check if your client is ready to make a decision:

- Do they have questions?
- Do they need more time?
- Do they want to make the decision themselves, or would they prefer to leave it to a healthcare provider or family member?
- Formulate the decision together and write it down.
- Outline the steps to be taken and who will be responsible.

**Helpful Phrases:**

- "Are you ready to make a decision?"
- "Do you need more time?"
- "Do you have any additional questions?"

**Step 6: Evaluation**

Discuss the decision made:

- Is it clear what the decision is?
- Can your client explain it in their own words?
- Is your client satisfied with their decision? If not, return to an earlier step.
- If yes, create a plan that aligns with the decision.

**Helpful Phrases:**

- "Can you tell me in your own words what we’ve agreed upon?"
- "Are you satisfied with the decisions we made?"
- "Would you like to revisit the decision?"
- "Do you need more time to think about it?"
- "What will we agree upon moving forward?"

**Link of the elearning:**

https://nl.dialoguetrainer.app/join/SQWMUCHV
