# Supplementary material for: Person-centered shared decision-making and data-informed district nursing care to enhance independence: Protocol for a feasibility study
Source: Int J Nurs Stud Adv. 2026 Jun 1;11:100569. doi: 10.1016/j.ijnsa.2026.100569 (PMC13266195; doi:10.1016/j.ijnsa.2026.100569)
Supplement: Supplementary file 3 [file mmc3.docx]

**Progress Intervention Group Measurement Point T1: Healthcare Providers**

Thank you for answering the questions on behalf of your team. Some questions relate to the implementation of the Data Nurse intervention by the team, and some focus on the tasks of the project leader or data ambassador. Completing the survey will take approximately 15 minutes. Your team has now been involved in the study for one month. Next week, we will have discussions with all participating project leaders and data ambassadors about the progress. The answers to these questions will be topics covered in that discussion.
We kindly ask you to provide honest answers. Your responses will be treated confidentially. During the progress discussions, it will not be disclosed which team answered what.
For multiple-choice questions, multiple answers may be selected.

**Participation**

1. How many clients were approached for participation in the study?
2. How many clients were not approached? What was the reason for this?
3. How many clients actually participated in the study?
4. What are the reasons that clients do not participate or drop out early?
   1. Too much work
   2. Too difficult
   3. Don't know
   4. Other, namely: ______________

**Logbook and Castor**

1. How many participating clients have been registered in the logbook?
2. How many participating clients were not registered in the logbook, and what was the reason?
   Number: ______________
   Reason: ______________
3. How many participating clients have been registered in Castor?
4. How many participating clients were not registered in Castor, and what was the reason?
   Number: ______________
   Reason: ______________

**Shared Decision-Making Tool (Samen Beslis Hulp)**

1. When was the Shared Decision-Making tool offered to clients?
   1. Before the intake or evaluation
   2. During the intake or evaluation
   3. After the intake or evaluation
   4. Other, namely: ______________
2. How often was the Shared Decision-Making tool used to create or evaluate the care plan?
3. What are the reasons within the team for not using the Shared Decision-Making tool?
   1. The tool is not considered a good questionnaire
   2. The client does not fill out the tool
   3. It was given too late
   4. Other, namely: ______________
4. What are the reasons participating clients do not use the Shared Decision-Making tool?
   1. Do not know the purpose of the tool
   2. Do not know how to use the tool (e.g., find it too difficult)
   3. Do not support the tool
   4. Did not receive it
   5. Other, namely: ______________

**E-learning**

1. If the e-learning was not completed, what was the reason?
   1. Did not receive an invitation with a link to the e-learning
   2. Too difficult overall
   3. Some topics were too difficult
   4. The virtual patient did not appeal
   5. Internet problems
   6. No time
   7. Not necessary: I already know enough about shared decision-making
   8. Other, namely: ______________
2. Is there a need for additional support regarding the e-learning?
   1. Yes
   2. No

**Workshop "Learning from Data" (given by the data ambassador within the team)**

1. Date the workshop was given to the team: ______________
2. The workshop has not yet been given
3. Reason, if not given: ______________
4. Can you proceed with the dashboard in the coming months?
   1. No, I would like more explanation from the data ambassador in my team on: ______________
   2. No, I have questions for the researchers about: ______________
   3. Yes, the workshop was sufficient for now
5. What rating does the data ambassador give the train-the-trainer workshop on data/dashboard given by the researcher?
   1 2 3 4 5 6 7 8 9 10
6. What rating does the data ambassador give themselves for the workshop they gave to the team on data/dashboard?
   1 2 3 4 5 6 7 8 9 10

**The Dashboard**

1. How many nurses (who attended the workshop) have gained access to the dashboard?
2. How often has the team consulted the dashboard to gain insights into client independence?
3. When is the dashboard consulted?
   1. During team meetings with the whole team
   2. During internal discussions with colleagues in the team
   3. During discussions with other disciplines
4. How often have changes been made to care plans after team meetings? (e.g., changes to care plans or care allocation)
5. How often was the dashboard inaccessible? What were the reasons for this?

**Progress Intervention Group Measurement Point T2: Healthcare Providers**

Thank you for answering the questions on behalf of your team. Some questions relate to the implementation of the Data Nurse intervention by the team, and others focus on the tasks of the project leader or data ambassador. Completing the survey will take approximately 15 minutes. Your team has now been participating in the study for two months. Next week, we will meet with all participating project leaders and data ambassadors to discuss progress. The answers to these questions will be topics covered in that discussion.
We kindly ask you to provide honest responses. Your answers will be treated confidentially. During the progress discussions, it will not be revealed which team provided which answers.
For multiple-choice questions, multiple answers may be selected.

**Participation**

1. How many clients are still participating in the study?
2. How many participating clients have discontinued care?
3. How many clients who discontinued care received a second questionnaire?
4. How many clients who discontinued care did not receive a second questionnaire?
   Number: ______________
   Reason: ______________
5. How many clients received a second questionnaire with the same registration number as the first one?
6. How is the process of linking registration numbers working?
7. What are the reasons if the registration number did not match the first questionnaire?
   1. The list with registration numbers was unclear
   2. The list with registration numbers was lost
   3. Other, namely: ______________
8. How useful is the logbook for linking clients to the correct registration number? Please rate on a scale from 1 to 10:
   1 2 3 4 5 6 7 8 9 10
9. What are the reasons clients drop out prematurely?
   1. Too much work
   2. Too difficult
   3. Don't know
   4. Other, namely: ______________

**Shared Decision-Making**

1. How many healthcare providers in the team apply the steps of Shared Decision-Making (as learned in the e-learning) in daily practice?
2. If yes, when are the steps applied?
   1. During intake and evaluations
   2. During care provision
   3. Other, namely: ______________
3. Which steps are considered applicable?
   1. Preparation of the client and healthcare provider and introduction to home care methods
   2. Discussing the client’s life goals and values
   3. Choosing the care goal with the client
   4. Indicating available options to achieve the care goal
   5. Deciding which care will be provided
   6. Evaluating the decision-making process and the decision
4. Which steps are considered difficult?
   1. Preparation of the client and healthcare provider and introduction to home care methods
   2. Discussing the client’s life goals and values
   3. Choosing the care goal with the client
   4. Indicating available options to achieve the care goal
   5. Deciding which care will be provided
   6. Evaluating the decision-making process and the decision
5. What are the reasons for not applying Shared Decision-Making in practice?
   1. The steps are (still) not mastered
   2. The steps are unclear
   3. The steps are not necessary
   4. The client and/or caregiver cannot make decisions on their own
   5. No time
   6. Other, namely: ______________
6. How useful are the steps of Shared Decision-Making in daily practice? Please rate on a scale from 1 to 10:
   1 2 3 4 5 6 7 8 9 10

**The Dashboard**

1. How many nurses (who attended the workshop) have gained access to the dashboard?
2. How often has the team consulted the dashboard to gain insights into client independence?
3. When is the dashboard consulted?
   1. During team meetings with the whole team
   2. During internal discussions with colleagues in the team
   3. During discussions with other disciplines
4. How often have changes been made to care plans after team meetings? (e.g., changes to care plans or care allocation)
5. How often was the dashboard inaccessible? What were the reasons for this?
6. How useful is the dashboard in daily practice? Please rate on a scale from 1 to 10:
   1 2 3 4 5 6 7 8 9 10

**Progress Intervention Group Measurement T3: Healthcare Providers**

Thank you for answering these questions on behalf of your team. Some questions focus on the implementation of the Data Nurse intervention by your team, while others concern the responsibilities of the team leader or data ambassador. Completing this will take approximately 15 minutes. You have now participated in the study for three months. Next week, we will meet with all participating team leaders and data ambassadors to discuss progress. The answers to these questions will be topics of discussion during that meeting.

We ask you to answer honestly. We will treat your responses confidentially, and during the progress meetings, it will not be disclosed which team provided which answers. Multiple answers are possible for the multiple-choice questions.

**Participation**

1. How many clients are still participating in the study?
2. How many participating clients have exited care?
3. How many clients who exited care received a second questionnaire?
4. How many clients received a questionnaire with the same registration number as the first questionnaire?
5. Is additional support from the researchers needed for client registration and linking the second questionnaire?
   - Yes
   - No
6. If yes, what support is needed?
   - Online explanation for the team leader
   - Online explanation for the team
   - Other, please specify:

**Shared Decision-Making (Samen Beslissen)**

1. How many healthcare providers in the team apply the steps of Shared Decision-Making, as taught in the e-learning, in daily practice?
2. If yes, when are the steps applied?
   - During intakes and evaluations
   - During care provision
   - Other, please specify:
3. Which steps are considered applicable in practice?
   - Preparing the client and healthcare provider and introducing the district nursing process
   - Discussing the client’s life goals and values
   - Choosing care goals with the client
   - Indicating available options to achieve the care goal
   - Deciding which care will be provided
   - Evaluating the decision-making and the decision
4. Which steps are considered challenging to apply?
   - Preparing the client and healthcare provider and introducing the district nursing process
   - Discussing the client’s life goals and values
   - Choosing care goals with the client
   - Indicating available options to achieve the care goal
   - Deciding which care will be provided
   - Evaluating the decision-making and the decision
5. What are the reasons for not applying Shared Decision-Making in practice?
   - The steps are not yet mastered
   - The steps are unclear
   - The steps are unnecessary
   - The client and/or caregiver cannot make decisions independently
   - No time for it
   - Other, please specify:
6. Is additional support from the researchers needed regarding Shared Decision-Making?
   - Yes
   - No
7. If yes, what support is needed?
   - Online explanation for the team leader
   - Online explanation for the team
   - Other, please specify:

**Dashboard**

1. How many nurses (who attended the workshop) have gained access to the dashboard?
2. How often has the team consulted the dashboard to gain insights into client self-sufficiency?
3. When is the dashboard consulted?
   - During team meetings with the whole team
   - During internal discussions with team colleagues
   - During discussions with other disciplines
4. How often have changes been made to care plans or care allocation following team meetings?
5. How often was the dashboard inaccessible? What were the reasons for this?
6. Is additional support from the researchers needed regarding the dashboard?
   - Yes
   - No
7. If yes, what support is needed?
   - Online explanation for the team leader
   - Online explanation for the team
   - Other, please specify:

**Progress Intervention Group Measurement T4: Healthcare Providers**

Thank you for answering these questions on behalf of your team. Some questions focus on the implementation of the Data Nurse intervention by your team, while others concern the responsibilities of the team leader or data ambassador. Completing this will take approximately 15 minutes. You have now participated in the study for four months. Next week, we will meet with all participating team leaders and data ambassadors to discuss progress. The answers to these questions will be topics of discussion during that meeting.

We ask you to answer honestly. We will treat your responses confidentially, and during the progress meetings, it will not be disclosed which team provided which answers. Multiple answers are possible for the multiple-choice questions.

**Participation**

1. How many clients are still participating in the study?
2. How many participating clients have exited care?
3. How many clients who exited care received a second questionnaire?
4. How many clients received a questionnaire with the same registration number as the first questionnaire?

**Shared Decision-Making (Samen Beslissen)**

1. How many healthcare providers in the team apply the steps of Shared Decision-Making, as taught in the e-learning, in daily practice?
2. If yes, when are the steps applied?
   - During intakes and evaluations
   - During care provision
   - Other, please specify:
3. Which steps are successfully applied in practice?
   - Preparing the client and healthcare provider and introducing the district nursing process
   - Discussing the client’s life goals and values
   - Choosing care goals with the client
   - Indicating available options to achieve the care goal
   - Deciding which care will be provided
   - Evaluating the decision-making and the decision
4. What positive effects have the steps of Shared Decision-Making had on care delivery?
   - Conversations are more structured
   - We reach decisions with the client more quickly
   - Clients better understand that district nursing focuses on self-sufficiency and participation in care
   - Clients can better express what care they need
   - The client’s care goals are better defined in the care plan
   - Care is more aligned with the client’s wishes than before
   - Other, please specify:

**The Dashboard**

1. How many nurses (who attended the workshop) have gained access to the dashboard?
2. How often has the team consulted the dashboard to gain insights into client self-sufficiency?
3. When is the dashboard consulted?
   - During team meetings with the whole team
   - During internal discussions with team colleagues
   - During discussions with other disciplines
4. How often have changes been made to care plans or care allocation following team meetings?
5. How often was the dashboard inaccessible? What were the reasons for this?
6. What positive effects has the dashboard had on care delivery?
   - We use information about self-sufficiency from the client’s file more effectively
   - We have better insights into self-sufficiency
   - We have more insights into which interventions we can use to improve self-sufficiency
   - We have better insights into the impact of our interventions
   - We can provide clients with insights into their own self-sufficiency
   - Other, please specify:

**Progress Intervention Group Measurement T5: Healthcare Providers**

Thank you for answering these questions on behalf of your team. Some questions focus on the implementation of the Data Nurse intervention by your team, while others concern the responsibilities of the team leader or data ambassador. Completing this will take approximately 15 minutes. You have now participated in the study for five months. You have completed the last questionnaire, and all participating clients have received and completed the final questionnaire. Next week, we will meet with all participating team leaders and data ambassadors to evaluate the intervention. The answers to these questions will be topics of discussion during that meeting.

We ask you to answer honestly. We will treat your responses confidentially, and during the progress meetings, it will not be disclosed which team provided which answers. Multiple answers are possible for the multiple-choice questions.

**Participation**

1. How many clients will participate until the end of the study?
2. Did all participating clients receive the second questionnaire?
3. How many clients received a questionnaire with the same registration number as the first questionnaire?

**Shared Decision-Making (Samen Beslissen)**

1. How many healthcare providers in the team apply the steps of Shared Decision-Making, as taught in the e-learning, in daily practice?
2. If yes, when are the steps applied?
   - During intakes and evaluations
   - During care provision
   - Other, please specify:
3. Which steps are successfully applied in practice?
   - Preparing the client and healthcare provider and introducing the district nursing process
   - Discussing the client’s life goals and values
   - Choosing care goals with the client
   - Indicating available options to achieve the care goal
   - Deciding which care will be provided
   - Evaluating the decision-making and the decision
4. What does the team need to continue using the steps of Shared Decision-Making?
   - Annual refresher course
   - Continued access to the Dialogue Trainer e-learning
   - Mainly continuing to apply Shared Decision-Making in practice
   - Other, please specify:

**The Dashboard**

1. How often has the team consulted the dashboard to gain insights into client self-sufficiency?
2. When is the dashboard consulted?
   - During team meetings with the whole team
   - During internal discussions with team colleagues
   - During discussions with other disciplines
3. How often have changes been made to care plans or care allocation following team meetings?
4. How often was the dashboard inaccessible? What were the reasons for this?
5. What positive effects has the dashboard had on care delivery?
   - We use information about self-sufficiency from the client’s file more effectively
   - We have better insights into self-sufficiency
   - We have more insights into which interventions we can use to improve self-sufficiency
   - We have better insights into the effect of our interventions
   - We can provide clients with insights into their own self-sufficiency
   - Other, please specify:
6. What does the team need to continue using the dashboard?
   - Annual refresher course
   - Mainly continuing to apply it in practice
   - Other, please specify:
