# Supplementary material for: Person-centered shared decision-making and data-informed district nursing care to enhance independence: Protocol for a feasibility study
Source: Int J Nurs Stud Adv. 2026 Jun 1;11:100569. doi: 10.1016/j.ijnsa.2026.100569 (PMC13266195; doi:10.1016/j.ijnsa.2026.100569)
Supplement: Supplementary file 4 [file mmc4.docx]

Questionnaire: Shared Decision-Making with Older Adults on Interventions to Promote Independent Functioning

In district nursing, we make many decisions with older adults about interventions to promote independent functioning. This often happens during an intake or evaluation. The following questions are about making these decisions in daily practice.

Informing

1. I inform my clients what "Shared Decision-Making" means in district nursing.

Always | Often | No Opinion | Sometimes | Never

0 | 0 | 0 | 0 | 0

2. I explain to my clients that district nursing decides together with them which interventions are needed to live independently at home.

Always | Often | No Opinion | Sometimes | Never

0 | 0 | 0 | 0 | 0

Conversation Structure

1. I explain to my clients beforehand how decisions will be made.

Always | Often | No Opinion | Sometimes | Never

0 | 0 | 0 | 0 | 0

2. My client determines the structure of the conversation.

Always | Often | No Opinion | Sometimes | Never

0 | 0 | 0 | 0 | 0

Patient SDM Preparatory Tool

1. I discuss the completed Patient SDM Preparatory Tool with my clients.

Always | Often | No Opinion | Sometimes | Never

0 | 0 | 0 | 0 | 0

### Information from the Patient SDM Preparatory Tool

1. I discuss: Experienced health (pain, memory, ADLs, social factors, etc.)

Always | Often | No Opinion | Sometimes | Never

0 | 0 | 0 | 0 | 0

2. I discuss: What the client considers important to discuss.

Always | Often | No Opinion | Sometimes | Never

0 | 0 | 0 | 0 | 0

3. I discuss: What the client thinks needs to happen to improve their quality of life.

Always | Often | No Opinion | Sometimes | Never

0 | 0 | 0 | 0 | 0

4. I discuss: What the client wants to reduce in terms of distress or hopes to regain.

Always | Often | No Opinion | Sometimes | Never

0 | 0 | 0 | 0 | 0

5. I discuss: Information from the informal caregiver.

Always | Often | No Opinion | Sometimes | Never

0 | 0 | 0 | 0 | 0

6. I discuss: With whom the client would like to make decisions.

Always | Often | No Opinion | Sometimes | Never

0 | 0 | 0 | 0 | 0

7. I discuss: Important life goals and values (perspective on life, what brings the client joy, hopes, or fears).

Always | Often | No Opinion | Sometimes | Never

0 | 0 | 0 | 0 | 0

Discussing Goals and Options

1. I formulate concrete care goals together with the client and/or their family members.

Always | Often | No Opinion | Sometimes | Never

0 | 0 | 0 | 0 | 0

2. I discuss the pros and cons of possible care options with the client and/or family members.

Always | Often | No Opinion | Sometimes | Never

0 | 0 | 0 | 0 | 0

3. I ask the client and/or family members if they see any other options or could take on any aspects themselves (e.g., lifestyle interventions, social interventions).

Always | Often | No Opinion | Sometimes | Never

0 | 0 | 0 | 0 | 0

4. I discuss with the client that we are making the decision together.

Always | Often | No Opinion | Sometimes | Never

0 | 0 | 0 | 0 | 0

5. I check whether the client has understood the decision made, for example by using the "teach-back method."

Always | Often | No Opinion | Sometimes | Never

0 | 0 | 0 | 0 | 0

Outcome of the Patient SDM Preparatory Tool

1. What has shared decision-making with the Patient SDM Preparatory Tool yielded for your clients so far?

---

2. What has shared decision-making with the Patient SDM Preparatory Tool yielded for you as a healthcare provider?

---
