# Supplementary material for: Person-centered shared decision-making and data-informed district nursing care to enhance independence: Protocol for a feasibility study
Source: Int J Nurs Stud Adv. 2026 Jun 1;11:100569. doi: 10.1016/j.ijnsa.2026.100569 (PMC13266195; doi:10.1016/j.ijnsa.2026.100569)
Supplement: Supplementary file 5 [file mmc5.pdf]

Niet-WMO toetsingscommissie
Amsterdam UMC
metc@amsterdamumc.nl

Datum: Amsterdam, 3 juni 2025
Ons kenmerk: 2024.0956
Betreft: Niet-WMO verklaring

Geachte prof. dr. B.M. Buurman-van ES,

De Niet-WMO toetsingscommissie Amsterdam UMC heeft uw onderzoek **Pilot studie Data Nurse: optimaliseren van de zelfredzaamheid bij ouderen met wijkverpleging** besproken in de vergadering van 3-12-2024.

De commissie is van oordeel dat bovengenoemd onderzoek niet onder de reikwijdte van de Wet Medisch-wetenschappelijk Onderzoek met mensen (WMO) valt. De reden hiertoe is dat er sprake is van medisch-wetenschappelijk onderzoek, maar personen die meedoen niet worden onderworpen aan handelingen en er geen bepaalde gedragswijze wordt opgelegd.

De Niet-WMO toetsingscommissie Amsterdam UMC is, op grond van het protocol en de aangeleverde stukken, van oordeel dat de opzet van uw onderzoek voldoet aan de vereisten die voortvloeien uit onderstaande wet- en regelgeving die (mogelijk) van toepassing is:

- ECTR, MDR of IVDR
- WGBO (Wet op de Geneeskundige Behandelingsovereenkomst);
- AVG (Algemene Verordening Gegevensbescherming), zie https://autoriteitpersoonsgegevens.nl/nl/onderwerpen/avg-europese-privacywetgeving;
- Gedragscode Gezondheidsonderzoek, zie www.coreon.org;
- Biobanken: Reglement Toetsing Biobanken Amsterdam UMC, zie https://metc.amsterdamumc.org/nieuwsberichten/vervolg-onderzoek-met-lichaamsmateriaal-biobank/

De verklaring is gebaseerd op de volgende documenten:

Type	Versienummer	Documentdatum	Additionele informatie
A1. Aanbiedingsbrief	2	05-11-2024	
C1. Onderzoeksprotocol	4	02-04-2025	
C1. Onderzoeksprotocol	4	02-04-2025	TC
E1/E2. Informatiebrief en toestemmingsformulier	3	11-03-2025	zorgverleners interventiegroep

E1/E2. Informatiebrief en toestemmingsformulier	3	11-03-2025	TC
E1/E2. Informatiebrief en toestemmingsformulier	3	11-03-2025	cliënten interventiegroep
E1/E2. Informatiebrief en toestemmingsformulier	3	11-03-2025	TC
E1/E2. Informatiebrief en toestemmingsformulier	2	11-03-2025	zorgverleners controlegroep
E1/E2. Informatiebrief en toestemmingsformulier	2	11-03-2025	TC
E1/E2. Informatiebrief en toestemmingsformulier	2	11-03-2025	cliënten controlegroep
E1/E2. Informatiebrief en toestemmingsformulier	2	11-03-2025	TC
E1/E2. Informatiebrief en toestemmingsformulier	5	22-05-2025	zorgverleners interventiegroep
E1/E2. Informatiebrief en toestemmingsformulier	5	22-05-2025	zorgverleners interventiegroep TC
E1/E2. Informatiebrief en toestemmingsformulier	4	22-05-2025	zorgverleners controlegroep
E1/E2. Informatiebrief en toestemmingsformulier	4	22-05-2025	zorgverleners controlegroep TC
F1. Vragenlijsten	1	25-10-2024	zorgverleners controlegroep
F1. Vragenlijsten	1	25-10-2024	zorgverleners controlegroep
F1. Vragenlijsten	1	25-10-2024	patiënten controlegroep
F1. Vragenlijsten	1	25-10-2024	patiënten interventiegroep
F1. Vragenlijsten	1	25-10-2024	patiënten interventiegroep
F1. Vragenlijsten	1	25-10-2024	zorgverleners interventiegroep
F1. Vragenlijsten	1	25-10-2024	zorgverleners interventiegroep
F1. Vragenlijsten	2	06-02-2025	Zorgverleners interventiegroep
F1. Vragenlijsten	2	06-02-2025	Zorgverleners interventiegroep/TC
F1. Vragenlijsten	2	06-02-2025	Zorgverleners interventiegroep
F1. Vragenlijsten	2	06-02-2025	Zorgverleners interventiegroep/TC
F1. Vragenlijsten	ontvangen d.d.	06-02-2025	cilenten controlegroep
F1. Vragenlijsten	ontvangen d.d.	06-02-2025	clienten interventiegroep
F1. Vragenlijsten	ontvangen d.d.	06-02-2025	zorgverleners interventiegroep
F1. Vragenlijsten	ontvangen d.d.	06-02-2025	Cliënten interventiegroep meetmoment 1 en 2
K10. Overige informatie	1	06-02-2025	screenshot dashboard

To whom it may concern

On behalf of the non-WMO Committee of the Medical Ethics Review Committee of Amsterdam University Medical Centers we are pleased to confirm that the Medical Research Involving Human Subjects Act (WMO) does not apply to the above mentioned study. This committee is supervised by the Medical Ethics Review committee and authorised by the board of directors of Amsterdam UMC.

The Medical Ethics Review Committee of Amsterdam University Medical Centers is registered with the US Office for Human Research Protections (OHRP) as IRB00013752. The FWA number assigned to Amsterdam UMC is FWA00032965.

Met vriendelijke groet,
namens de Niet-WMO toetsingscommissie Amsterdam UMC,

prof. dr. J.A.M. van der Post, voorzitter

Deze brief is ongetekend. Indien u een getekend exemplaar wilt ontvangen, vernemen wij dit graag.
