# Supplementary material for: Person-centered shared decision-making and data-informed district nursing care to enhance independence: Protocol for a feasibility study
Source: Int J Nurs Stud Adv. 2026 Jun 1;11:100569. doi: 10.1016/j.ijnsa.2026.100569 (PMC13266195; doi:10.1016/j.ijnsa.2026.100569)
Supplement: Supplementary file 6 [file mmc6.pdf]

Beste leden van de programmacommissie,

Wij willen de referenten hartelijk danken voor hun kritische reflectie en feedback.
De referenten geven aan dat ons project zeer relevant is voor de wijkverpleging met potentie voor internationale disseminatie. De referenten waarderen de focus op shared decision making en independence (zelfredzaamheid), en het idee dat de promovendi uit de praktijk komen waar het onderzoek zal plaatsvinden.
Een ander sterk punt dat is genoemd is de sterke onderzoeksgroep en internationale samenwerking met Omaha System expert prof. Karen Monsen en prof. Glyn Elwyn van het goal-based framework.

De rol van patiënten en het betrekken van het patiëntenperspectief is sterk ingebed in onze aanvraag. Echter was dit mogelijk niet zo expliciet beschreven of duidelijk gemaakt aangezien twee referenten aangaven dat dit punt nog ontbreekt. Patiënten en ouderenorganisatie KBO-PCOB is sterk vertegenwoordigd in ons project en we bespreken dit punt bij 2.3 in onderstaande wederhoor.

Wij zijn ervan overtuigd dat we alle punten van de referenten hebben beantwoord. Indien er aanvullende vragen zijn schroom dan niet om met ons contact op te nemen.

Met vriendelijke groeten,

Prof. dr. Bianca Buurman and dr. Nienke Bleijenberg

2.1 Objective, problem definition and assignment [Excellent, Good, Sufficient]

Reviewer **R.2021.157** addressed the following three points: 1) it would be helpful if there were specific research questions 2) there is no evidence presented regarding current methods for service improvement in district nursing 3) the application does not provide any details of literature searches they have already conducted in order to demonstrate that this study meets an evidence gap or the extent of the evidence likely to be available for example a scoping review. The other reviewers have no specific comments.

Reactie:
1) Wij hebben er voor gekozen om doelstellingen (aims) te formuleren in plaats van onderzoeksvragen. Deze doelstellingen kunnen eenvoudig omgezet worden naar vraagstellingen. Bijvoorbeeld aim 1: What does independence mean to older adults receiving district nursing and their informal care givers and how can independence be enhanced by the district nursing team on a population and individual level?
2) Zover wij weten is er op dit moment geen literatuur beschikbaar over het ontwikkelen van een learning health care system in de wijkverpleging. In andere gebieden zoals de geestelijke gezondheidszorg, zijn enkele voorbeelden beschreven waarin een data-gedreven benadering is uitgewerkt en toegepast. Bijvoorbeeld in de studie van Velupillai et al, 2019, waarin het voorspellen en voorkomen van suicide gedrag is onderzocht met behulp van meerdere datasets. Afgelopen jaren heeft Karen Monsen et al verschillende papers gepubliceerd over het gebruik van de Omaha System data ten behoeve van kwaliteitsverbetering in de klinische praktijk. Echter, tot op heden zijn er geen studies bekend waarin de Omaha System data is gebruikt in een zogeheten feedback loop om teams wijkverpleging te faciliteren in het leren werken met deze data in de praktijk. Dat is precies de reden waarom onze studies in potentie veel impact kunnen genereren zowel nationaal als internationaal.
3) De evidence ten aanzien van effectiviteit van de wijkverpleging is schaars (Jarrin et al, 2019). Recent heeft een van onze promovendi een systematische literatuurstudie afgerond naar de effectiviteit en uitkomsten in de wijkverpleging en concludeerde dat de interventies zeer heterogeen zijn in termen van componenten en op patientenuitkomsten, waaronder independence (zelfredzaamheid) (Submitted). Daarnaast zijn er verschillende papers gepubliceerd over het gebruik van de Omaha System data die cliëntenkenmerken en behoeften beschrijven (Topaz et al., 2014), meten, evalueren en verbeteren van verpleegkundige interventies (Monsen et al., 2017; Gao et al., 2019) en evalueren en voorspellen van patientenuitkomsten (Monsen et al., 2012; Macieira et al., 2017) in de wijkverpleging. Ons onderzoeksvoorstel zal daarom bijdragen aan het verder verstevigen van de huidige kennisontwikkeling op dit domein. In ons voorstel starten om die reden in werkpakket 1 met twee scoping-reviews en een exploratieve mixed-methods studie om het kennishiaat te slechten.

2.2. Strategy [Excellent, Good, Sufficient]

R.2021.159 mentioned that: 'independence' will need to be clearly defined at the start of the project as patients and nurses' perceptions on what it is, may be very different! Similarly, shared-decision making must be clearly defined so that common goals and objectives are achievable.

Reactie:

Wij zijn het eens dat independence (zelfredzaamheid) helder gedefinieerd moet worden bij de start van het project. Op basis van de literatuur, en op basis van de perceptie van patiënten en verpleegkundigen, het concept independence wordt in WP 1 in deel 1.3 uitgewerkt. Als startpunt gebruiken wij het model van Machteld Huber rondom positieve gezondheid (Huber et al, 2011). Wij zijn ons ervan bewust dat de perceptie van patiënten en verpleegkundigen kan verschillen. Daarom focussen we ons op "what matters to you" in plaats van "what is the matter".
Shared decision making (SDM), is gedefinieerd als 'a process in which decisions are made in a collaborative way, where trustworthy information is provided in accessible formats about a set of options, typically in situations where the concerns, personal circumstances, and contexts of patients and their families play a major role in decisions' (Elwyn et el 2017). In dit voorstel wordt de definitie aangepast naar de setting van de wijkverpleging.

R.2021.157: 1) Again it would be helpful to have the tasks (research projects) framed in terms of research questions. 2)The researchers have already specified the intervention (an education intervention) to pilot in WP 4. There does not seem to be any exploration prior to WP 4 a to district nurses views as to the acceptability of such intervention on this topic, perhaps this should be considered early on in this study? 3) Moreover, we will assess the experiences of patients and professionals with this strategy." but the detail is of the nurses experience and there is no detail as to how patients' experiences will be captured. I wonder if there should also be some attention paid to patients' views in wp1.

Reactie:

1)Wij hebben gekozen om doelstellingen (aims) te formuleren in plaats van onderzoeksvragen, zie onze reactie bij 2.1.
2)Een onderdeel van onze interventie bestaat inderdaad uit een training. Wij zijn het eens met de reviewer dat we eerder de acceptatie bij de verpleegkundigen en verzorgenden in de wijkverpleging kunnen exploreren. Wij zullen dit nagaan tijdens de behoefteanalyse in werkpakket 1. Daarnaast brengen wij de barrières en bevorderende factoren in kaart ten aanzien het leren werken met data. Tijdens de focusgroepen zullen we expliciet hun mening vragen ten aanzien van de trainingsinterventie.
3)Patiëntenervaringen en perspectief is zoals beschreven al meegenomen in werkpakket 1. Patiëntenervaringen en perspectieven op het gebied van persoonlijke doelen zijn meegenomen in de scoping review, zie taak 1.1. Daarnaast gaan we in werkpakket 1 met 50 ouderen in gesprek ten aanzien van het doelen stellen en hoe zij shared decision making (samen beslissen) ervaren.

2.3 Knowledge transfer [Excellent, Good, Good]

R.2021.159: It may be worthwhile having some patients on a steering group too.
Train the trainer is excellent but it must be done using a standardised protocol to ensure a homogenous approach in all centres.

Reactie:

Er is een patiëntenvertegenwoordiger vertegenwoordigd in de stuurgroep en projectgroep, zie bijlage 1. Dit is een lid van de KBO-PCOB. De KBO-PCOB is de grootste seniorenorganisatie in Nederland met ruim 250.00 leden. Hun doelstelling is om de positie van ouderen te verstevigen op lokaal, regionaal en nationaal niveau. Daarnaast bedanken wij de reviewer voor de suggestie dat we voor het onderdeel 'train de trainer' een gedetailleerd protocol moeten maken zodat dit verder verspreid en gedeeld kan worden. Wij nemen deze suggestie graag ter harte.

R.2021.157: There is no mention of the involvement of patients and/or patient organizations.

Reactie:

Het betrekken van patiënten en patiëntenorganisaties vinden wij zeer belangrijk en mogelijk hebben wij dit niet expliciet duidelijk gemaakt in ons voorstel. In onze stuurgroep en in onze expertgroep zijn namelijk wel degelijk patiënten en een patiëntenvertegenwoordiger van de KBO-PCOB opgenomen, zie bijlage 1. We hebben in bovenstaande reactie toegelicht wat de KBO-PCOB is.

2.4 Project group [Excellent, Excellent, Good]
R.2021.159: The importance of IT support and experts in IT for this cannot be under-estimated. Very clear instructions on what is required from IT will be required to ensure the databases is useful and different systems can 'talk' to each other (always a problem across healthcare).
Reactie:
Wij zijn het eens met de reviewer op bovengenoemd punt en wij zullen een uitgebreid handboek ontwikkelen om tot een bruikbare dataset te komen. Dit handboek kan ook gebruikt worden door andere organisaties die in de toekomst ook middels deze data-gedreven benadering willen gaan werken. Stichting Omaha System Support heeft op dit moment al goede ervaringen opgedaan op dit onderdeel, dus wij beginnen in dit project niet helemaal bij nul, maar borduren voort op hun kennis.

R.2021.157: The one aspect that does not seem well represented is the patient perspective.
Reactie:
Dit punt is geadresseerd in 2.3

2.5 Feasibility [Excellent, Excellent, Good]
R.2021.159: Yes, the project timelines are feasible but the risks are high for each WP to progress, they are dependent on the previous WP being completed. This will need close day to day project management. My only comment is that when analysing the work of the DNs, it would be really useful to add how much time each task takes and this would help with work allocation (especially as the number of older people increases). Also, the acuity of each task should be considered as part of the project and this would be really meaningful data and help with day-to-day workforce planning (often under-estimated and leads to unrealistic expectations).
Reactie:
Wij zijn ons ervan bewust dat het risicovol is dat ieder werkpakket van het voorafgaande werkpakket afhankelijk is. Echter, de PI en Co-PI zijn in elk werkpakket betrokken en zullen nauwkeurig de voortgang van het project monitoren. Beiden hebben veel ervaring met het managen van grote klinische studies in de eerstelijn waarin veel stakeholders betrokken zijn zoals ook het geval is in dit project. Dat geeft ons vertrouwen in de haalbaarheid van dit project. Verder vinden wij het een heel goed voorstel van de reviewer om na te gaan hoeveel tijd iedere taak in dit project van de professionals vraagt, met het idee dat dit goed inzichtelijk is wanneer we willen gaan opschalen of de benadering elders willen implementeren. Wij nemen dit mee in de feasibility study.

2.6 Overall quality assessment [Excellent, Good, Good]
R.2021.159: A very interesting project and well thought out. Shared decision-making is very important and although seen as a 'soft' approach, it is key on successful implementation of a service such as district nursing. Partnership with patients should be central to care and this project aims to do this. Good luck and I look forward to seeing the results. I really like the PhD candidates as part of the study- a great idea.
R.2021.158: The overall quality score is good - great national relevance to the project with potential for international dissemination to improve practice.
R.2021.157: This is a strong team developing and testing an educational intervention focused on enhancing the clinical practice of district nursing in promoting independence in their patients. It is an ambitious plan culminating in a pilot cluster randomized trial which will provide useful data but not necessarily evidence that points to implementation by others. However, findings from this and the other WPs will assist district nurses and district nursing service providers in better understanding how best to support older adults in regaining independence.
Reactie:
Wij bedanken de reviewers voor hun kritische, opbouwende reflectie en hun suggesties om ons voorstel verder aan te scherpen.

3. Budget [R, R, R]
Geen commentaar.

Referenties

- Velupillai, S., Hadlaczky, G., Baca-Garcia, E., Gorrell, G. M., Werbeloff, N., Nguyen, D., ... & Dutta, R. (2019). Risk assessment tools and data-driven approaches for predicting and preventing suicidal behavior. *Frontiers in psychiatry*, *10*, 36.
- Topaz M, Golfenshtein N, Bowles KH. The Omaha System: a systematic review of the recent literature. J Am Med Inform Assoc. 2014 Jan-Feb;21(1):163-70.
- Monsen KA, Vanderboom CE, Olson KS, Larson ME, Holland DE. Care Coordination From a Strengths Perspective: A Practice-Based Evidence Evaluation of Evidence-Based Practice. Res Theory Nurs Pract. 2017 Feb 1;31(1):39-55.
- Gao G, Kerr MJ, Lindquist RA, Chi CL, Mathiason MA, Monsen KA. Discovering Associations Among Older Adults' Characteristics and Planned Nursing Interventions Using Electronic Health Record Data. Res Theory Nurs Pract. 2019 Feb 1;33(1):58-80.
- Monsen KA, Swanberg HL, Oancea SC, Westra BL. Exploring the value of clinical data standards to predict hospitalization of home care patients. Appl Clin Inform. 2012 Nov 21;3(4):419-36. doi: 10.4338/ACI-2012-05-RA-0016. PMID: 23646088; PMCID: PMC3613033.
- Macieira, T. G., Smith, M. B., Davis, N., Yao, Y., Wilkie, D. J., Lopez, K. D., & Keenan, G. (2017). Evidence of progress in making nursing practice visible using standardized nursing data: a systematic review. In *AMIA Annual Symposium Proceedings* (Vol. 2017, p. 1205). American Medical Informatics Association.
- Elwyn G, Durand MA, Song J, et al. A three-talk model for shared decision making: multistage consultation process. BMJ (Clinical research ed) 2017; 359: j4891. 2017/11/08. DOI: 10.1136/bmj.j4891.
