# Supplementary material for: Person-centered shared decision-making and data-informed district nursing care to enhance independence: Protocol for a feasibility study
Source: Int J Nurs Stud Adv. 2026 Jun 1;11:100569. doi: 10.1016/j.ijnsa.2026.100569 (PMC13266195; doi:10.1016/j.ijnsa.2026.100569)
Supplement: Supplementary file 7 [file mmc7.pdf]

ZonMw stimuleert gezondheidsonderzoek en zorginnovatie

Laan van Nieuw Oost-Indië 334
2593 CE Den Haag
Postbus 93245
2509 AE Den Haag
Telefoon 070 349 51 11
Fax 070 349 51 00
www.zonmw.nl
info@zonmw.nl

Dossiernummer
80-86300-98-057
Ons kenmerk
2021/17800/ZONMW

Datum
24 juni 2021

Contactpersoon
Dineke Abels
Telefoon 070 349 54 66
verplegingenverzorging@zonmw.nl

Amsterdam UMC - locatie AMC
Raad van Bestuur
De heer Prof. dr. J.A. Romijn
Postbus 22660
1100 DD AMSTERDAM ZUIDOOST

Onderwerp
Subsidiebesluit over dossiernummer 10040022010003

Geachte heer Romijn,

ZonMw heeft een besluit genomen over uw subsidieaanvraag '*Data Driven Essential Care in District Nursing: improving patient outcomes and maintaining independence'*. In deze brief vindt u ons besluit en leest u wat u van ons kunt verwachten. Daarnaast leest u wat wij van u verwachten.

Besluit
ZonMw besluit tot honorering van uw subsidieaanvraag. In overeenstemming met de door u ingediende begroting, ontvangt u een subsidie van maximaal € 599.680,- voor de duur van maximaal 48 maanden. Dit bedrag is inclusief eventuele btw.

Onderbouwing besluit
De beoordelingscommissie van het programma Verpleging en Verzorging heeft positief geadviseerd over uw aanvraag. Het advies van de commissie is naar het oordeel van ZonMw op zorgvuldige wijze tot stand gekomen. ZonMw heeft dit advies overgenomen en haar besluit hierop gebaseerd.

Alle subsidieaanvragen zijn beoordeeld op kwaliteit en relevantie voor het programma en de subsidieoproep *Onderzoeksprojecten Verpleging en Verzorging – Essentiële zorg.*

Oordeel over relevantie
De commissie heeft het volgende relevantieoordeel over uw subsidieaanvraag gegeven: **zeer relevant**.

Het relevantieoordeel is op de volgende argumenten gebaseerd:
- Uw aanvraag sluit goed aan bij de doelstellingen van deze subsidieronde.
- Het gaat hier om een innovatief project waarin, passend bij de landelijke ontwikkelingen en gebruik makend van data, verbeteringen in de wijkverpleging en thuiszorg tot stand worden gebracht.
- Het betreft een ambitieus en grootschalig onderzoek dat goed aansluit op het thema essentiële zorg.

De commissie heeft het volgende oordeel over de kwaliteit van uw subsidieaanvraag: **goed**

Het kwaliteitsoordeel is op de volgende argumenten gebaseerd:
- De referenten zijn positief over dit project en de commissie sluit zich aan bij de referentenoordelen. De vragen en kritiekpunten van de referenten zijn in het wederhoor helder verwerkt dan wel weerlegd.
- De commissie ziet in de aanvraag de realisatie van combinatiebanen voldoende naar voren komen. U dient concreter uit te werken hoe de bestuurlijke inbedding en borging hiervan wordt vormgegeven.
- Bij de uitvoering van uw project wordt onder andere gewerkt aan het verstevigen van de infrastructuur voor toekomstig onderzoek. Hierbij vraagt de commissie u tijdens het project de evolutie vanuit de huidige situatie naar de toekomst vast te leggen zodat dit in de tijd gevolgd kan worden als leerschool voor andere consortia.
- Verder vraagt de commissie u om het management van de thuiszorgorganisaties meer te betrekken om deelname aan het project te blijven faciliteren. In uw aanvraag komt dit niet voldoende aan bod.
- Het cliëntenperspectief is gewaarborgd met een vertegenwoordiger van KBO in de stuurgroep en twee cliënten in de expertgroep. De commissie vindt dit wat mager en adviseert u waar mogelijk en zinvol, meer patiënten te betrekken bij de uitvoering van het project.

U dient bovenstaande punten, evenals de aanscherpingen die u heeft gemaakt in uw wederhoor, te betrekken bij de uitvoering van uw project en hier bij het eerste voortgangsverslag over te rapporteren.

Regelgeving en algemene voorwaarden
Op dit besluit is de volgende regelgeving van toepassing:
- De Algemene Wet Bestuursrecht
- De Algemene subsidiebepalingen ZonMw. U kunt deze nalezen op: www.zonmw.nl/subsidievoorwaarden
- Procedurebrochure voor aanvragers. De brochure kunt u downloaden op: https://www.zonmw.nl/nl/subsidies/hoe-werkt-subsidie-aanvragen. Bij punt 17 t/m 21 van de brochure leest u wat er gedurende het project van u wordt verwacht.

Specifieke voorwaarden
De subsidie wordt verleend onder de volgende (opschortende) voorwaarden:

- *Opschortende voorwaarde*
  Zoals in de subsidieoproep is beschreven, dient u een samenwerkingsovereenkomst aan te leveren. Zie hiervoor het ZonMw format. De overeenkomst dient getekend te worden door alle betrokken partijen en aan ZonMw te worden toegestuurd. Wij zien de samenwerkingsovereenkomst graag z.s.m. en uiterlijk **20 augustus 2021** tegemoet via email: verplegingenverzorging@zonmw.nl.
- ZonMw keert pas een voorschot uit als aan alle eisen voor het uitvoeren van het onderzoek is voldaan. Wij raden u aan eventuele procedures hiervoor tijdig te starten. Denk bijvoorbeeld aan een positief oordeel van een erkende medisch-ethische toetsingscommissie (METC), de Centrale Commissie Mensgebonden Onderzoek (CCMO), een projectvergunning van de Centrale Commissie Dierproeven (CCD), of een vergunning krachtens de Wet op het Bevolkingsonderzoek (WBO). U kunt bij de betreffende instanties nagaan of uw project dergelijke verklaringen of vergunningen nodig heeft.
  Wij wijzen u er op dat u zelf verantwoordelijk bent voor het tijdig aanvragen en in bezit hebben van deze documenten. Bij niet naleven

van deze voorwaarden kan ZonMw de verleende subsidie lager of op nihil vaststellen.

- *Open science – Datamanagement*
  Om data uit uw project ook in de toekomst herbruikbaar te laten zijn, dient u een datamanagementplan op te stellen. Informatie over hoe u dat moet doen, vindt u op de ZonMw website via www.zonmw.nl/en/research-and-results/fair-data-and-data-management/data-management-in-your-project/. Volg vanaf deze link de stappen in de procedure.
  Dien uiterlijk **3 maanden** na verzenddatum van deze brief uw datamanagementplan en (voorlopige) kerngegevens in. Beide kunt u sturen naar verplegingenverzorging@zonmw.nl Indien u geen dataverzameling opbouwt, kunt u dat melden bij het programmateam.
- *Open science - Open access*
  Alle publicaties die voortkomen uit wetenschappelijk onderzoek dat geheel of gedeeltelijk door ZonMw gesubsidieerd is dienen direct (zonder embargo) Open Access beschikbaar gesteld te worden, overeenkomstig met het ZonMw Open Access beleid.
  Aan een door ZonMw verleende subsidie is de uitdrukkelijke voorwaarde verbonden dat (tenzij in de subsidieoproep anders is bepaald) op de auteursversie van alle toekomstige publicaties voortkomend uit een ZonMw subsidie een onherroepelijke, niet-exclusieve CC BY licentie van toepassing is. U dient van het bestaan van deze licentie melding te maken aan elke partij met wie u in overleg treedt omtrent publicatie van (een deel van de) projectresultaten. ZonMw is te allen tijde gerechtigd aan derden melding te maken van deze licentie. Voor meer informatie over het ZonMw Open Access beleid zie: www.zonmw.nl/nl/over-zonmw/open-science-fair-data/open-access/.
- Indien u Open Access publicatiekosten heeft opgenomen in de projectbegroting dient u t.z.t. de naam van het journal en een bewijs van betaling Article Processing Charges te mailen naar verplegingenverzorging@zonmw.nl en openscience@zonmw.nl.'
- U dient te rapporteren over uw publicaties in ProjectNet en het voortgangs- en eindverslag.
- *Kennisbenutting*
  ZonMw wil weten wat er met de resultaten van uw project gebeurt. Geef in de voortgangsverslagen aan met wie u samenwerkt en welke verspreiding- en implementatieactiviteiten u onderneemt. Geef aan of de resultaten direct geïmplementeerd worden in de praktijk. Spelen de resultaten een rol bij het maken van beleid, onderwijs, een volgende stap in een wetenschappelijke carrière of vormen de resultaten een basis voor een nieuw project? U bent verplicht om ZonMw tot 4 jaar na afronding via ProjectNet te informeren over het gebruik van de resultaten en publicaties.

Integriteit
Het project dient op een goede en integere manier uitgevoerd te worden. Hiervoor gelden onder andere de volgende principes: eerlijkheid, zorgvuldigheid, transparantie, onafhankelijkheid en verantwoordelijkheid. Deze principes zijn vastgelegd in de Nederlandse gedragscode wetenschappelijke integriteit . Indien bij een door ZonMw gefinancierd project een (mogelijke) integriteitsschending plaatsvindt, dient ZonMw hier zonder uitstel van op de hoogte te worden gesteld. Alle relevant documenten over de integriteitsschending dienen aan ZonMw te worden overhandigd.

Start project
Het project dient uiterlijk **6 maanden** na de verzenddatum van deze brief te beginnen. Gaat het project later van start, dan vervalt de honorering van uw aanvraag. Hiervan kan alleen in bijzondere gevallen worden afgeweken.

Toelichting op hoogte subsidiebedrag
Van het subsidiebedrag is € 5.000,- gereserveerd voor open access publicatie kosten. Van de verschillende posten dient bij de eindafrekening een specificatie te worden aangeleverd.

Betaling van de subsidie
De subsidie zal via voorschotbedragen aan u worden overgemaakt. De betalingen zijn afhankelijk van de ontvangst en goedkeuring van voortgangsverslagen en de eindverantwoording van uw project. Als er aan alle door ZonMw gestelde voorwaarden is voldaan, ontvangt u rond de startdatum van het project het eerste voorschot.

Voortgangsverslag, netwerkbijeenkomsten en eindverantwoording
ZonMw wil graag op de hoogte blijven van de voortgang van uw project. Van het programmasecretariaat ontvangt in het derde kwartaal 2022 het verzoek een eerste voortgangsrapportage in te dienen. Rond die tijd wordt ook de eerste netwerkbijeenkomst georganiseerd. Deze heeft als doel kennis en ervaringen te delen en de onderlinge samenwerking te bevorderen. Ook kan er in de loop van het programma aan u gevraagd worden in samenwerking met ZonMw een netwerkbijeenkomst te organiseren.

Binnen 13 weken na afloop van uw project dient u een eindverantwoording in. Dit is een inhoudelijk eindverslag en de financiële eindverantwoording. De financiële verantwoording dient een specificatie van de verschillende posten te bevatten. Na ontvangst en goedkeuring van de eindverantwoording vindt de definitieve subsidievaststelling en afrekening plaats op basis van werkelijk gemaakte kosten.

Tussentijdse wijzigingen
Laat het ons weten als er tussentijds iets in uw plan of uw organisatie verandert. Verandering in de opzet, planning, begroting of organisatie kunnen gevolgen hebben voor uw subsidie. Pas na goedkeuring door ZonMw zijn eventuele wijzigingen toegestaan.

Wat verwachten wij nu van u?
ZonMw kan u een voorschot voor de eerste periode van uw project verstrekken. Hiervoor dient u binnen **4 weken** na de verzenddatum van deze brief:

- Bijgevoegd meldingsformulier ingevuld retour te sturen.
- Een Nederlandse publiekssamenvatting in ProjectNet in te vullen (maximaal 1000 tekens, inclusief spaties). ZonMw publiceert alle gehonoreerde projecten op haar website met een Nederlandse samenvatting in eenvoudige taal. Deze is bedoeld voor een breed geïnteresseerd publiek met verschillende achtergronden. Zie de schrijfwijzer op http://www.zonmw.nl/nl/over-zonmw/logo-huisstijl.

Dossiernummer
80-86300-98-057

Ons kenmerk
2021/17800/ZONMW

Datum
24 juni 2021

Vragen
Heeft u vragen? Neemt u dan gerust contact op met Dineke Abels, senior programmamanager. Dat kan via e-mail: verplegingenverzorging@zonmw.nl of via telefoonnummer: 070 349 54 66. Houd uw dossiernummer bij de hand zodat wij u snel kunnen helpen. Het oorspronkelijke nummer van uw aanvraag vervalt. Er geldt het nieuwe projectnummer: **10040022010003.**

Bezwaarclausule
Bent u het niet eens met dit besluit? U kunt tot 6 weken na de verzenddatum van deze brief bezwaar maken. Liever hebben wij dat u eerst contact met ons opneemt. Wij beantwoorden graag uw vragen over deze brief.
Blijft u het oneens? U kunt een bezwaarschrift sturen naar het bestuur van ZonMw, t.a.v. Commissie Bezwaarschriften ZonMw, Postbus 93 245, 2509 AE Den Haag. Meer informatie over bezwaar maken vindt u op de ZonMw website via www.zonmw.nl/signalerenklagenbezwaarmaken.

Ik feliciteer u met de honorering van uw subsidieaanvraag en wens u succes bij de uitvoering van uw project!

Met vriendelijke groet,
namens het bestuur van ZonMw,

Véronique Timmerhuis
Algemeen directeur

Bijlage(n)
Meldingsformulier start project

Kopie wordt per mail verzonden aan:
Prof. dr. B.M. Buurman
Dr. N. Bleijenberg
