# Supplementary material for: Person-centered shared decision-making and data-informed district nursing care to enhance independence: Protocol for a feasibility study
Source: Int J Nurs Stud Adv. 2026 Jun 1;11:100569. doi: 10.1016/j.ijnsa.2026.100569 (PMC13266195; doi:10.1016/j.ijnsa.2026.100569)
Supplement: Supplementary file 8 [file mmc8.pdf]

Subsidieprogramma / Subsidy programme	:	**Verpleging en Verzorging**
Dossiernummer / Dossier number	:	**80-86300-98-057**
Aanvrager / applicant	:	**Prof. dr. B.M. Buurman**
Projecttitel / Project title	:	**Data Driven Essential Care in District Nursing: improving patient outcomes and maintaining independence**
Beoordelingscode / Assessment code	:	**B.2021.003A0**

1. General information

Please read before reviewing the proposal.

Programme

The programme Nursing and Care is designed to enhance the professionalism of nurses, carers and nursing specialists, and is thus important for the attractiveness of the profession and the quality of care.

Call for proposals

The purpose of this call is the acquisition of five projects. These projects should contribute to the evidence base for nursing and/or care practices on the theme of essential care. The results and recommendations that emerge from the studies must have a good likelihood of being applied (in other words, they must be implementable in practice).

2. Criteria

Legenda: E (Excellent), G (Good), S (Sufficient), M (Moderate), U (Unsatisfactory)

2.1 Objective, problem definition and assignment

E	G	S	M	U
		X		

Consider the following factors:

- how clear and specific the objective is;
- how clear and verifiable the problem definition/assignment is and whether it is consistent with the objective, namely substantiating nursing and care activities, better patient outcomes, and improving the quality of care;
- the value added to existing knowledge or practice
- the theoretical or empirical evidence presented in support of the problem definition/assignment

Enter your considerations:

Thank you for the opportunity to review this research proposal focused on the practice of district nursing. As the co-applicants have stated this is a field of nursing with a relatively poorly developed evidence base.
The applicants provide “The overall goal of this proposal is to develop a learning health care system in district nursing and use this learning health care system to improve independence in older persons receiving district nursing.” There are five stated aims – the last being a more general one about Building and strengthening the national scientific and knowledge infrastructure in district nursing care. As this as, I understand it, is a research grant - I would have found it helpful if there were specific research questions.

I found the problem specification via an illustrative , fictionalized clinical case unusual in a research grant application. I think I understood that there was two main problems identified 1) that district nurses in the Netherlands are not currently provided with data from their electronic records that allows them to judge progress or otherwise on their agreed (with the patient) objectives and plans on achieving independence (I'm not sure if this meant from the DN service or from all sources of assistance) and 2) district nurses lack the evidence based knowledge in how to promote independence with their patients. Addressing these problems seems "consistent with the objective, namely substantiating nursing and care activities, better patient outcomes, and improving the quality of care.
While the applicants state their overall goal is to create a learning health care system there is no evidence presented regarding current methods for service improvement in district nursing.
The application does not provide any details of literature searches they have already conducted in order to

demonstrate that this study meets an evidence gap or the extent of the evidence likely to be available for example for the scoping review.

2.2 Strategy

E	G	S	M	U
		X		

Consider the following factors:

- clarity;
- adequacy in terms of problem definition/assignment;
- adequacy of chosen methods and analyses including theoretical and/or empirical substantiation;
- a power analysis if applicable:
- collection and analysis of the experiences from patients and their relatives;
- if there is a target group:
    - the way in which the strategy reflects the factors gender, age, ethnicity and/or other characteristics relevant to the objective;
    - degree of collaboration with intermediate and/or ultimate target group (the patient/client perspective).

Enter your considerations:

This is an ambitious, mixed methods study organized into 4 work packages with 3 or 4 tasks (projects) in each and finishing with a fifth work package of disseminating the findings. The strategy of using the MRC framework for the development and testing of complex interventions is appropriate.

Again it would have been helpful to have the tasks (research projects) framed in terms of research questions .

The researchers have already specified the intervention (an education intervention) to pilot in WP4.

There does not seem to be any exploration prior to WP4 as to district nurses views as to the acceptability of such an intervention on this topic - perhaps this should be considered early on in the study ?

The applicants state that "Moreover, we will assess
the experiences of patients and professionals with this strategy." but the detail is of the nurses experience and there is no detail as to how patients' experiences will be captured . I wonder if there should also be some attention paid to patients' views in wp1 ?

2.3 Knowledge transfer

E	G	S	M	U
	X			

Consider the following factors:

- nurses and carers are the end users of the results. All knowledge which stems from the research line must be accessible, applicable in practice, be of national relevance and lead to an improved quality of care.
- cooperation with knowledge centres and educational- and practical institutions
- the involvement of patients and/or patient organizations
- the knowledge will be incorporated into training programmes and curricula of educational establishments, in extra training and further education activities and, where possible, in guidelines/standards

Enter your considerations:

There is a very detailed and comprehensive plan for dissemination of findings to nurses , nurse organizations and educators.
There is no mention of the involvement of patients and/or patient organizations.
I particularly like the inclusion of funding for PhD students who will also be practicing district nurses.

2.4 Project group

E	G	S	M	U
	X			

Consider the following factors:
- relevant expertise concerning research, practice, education, implementation, and patient perspective;
- familiarity with area in question;
- prior activities and products

Enter your considerations:

This seems a very strong group of co-applicants with relevant expertise concerning research, practice, education, implementation, familiarity with area in question; prior activities and products.

The one aspect that does not seem well represented is the patient perspective.

2.5 Feasibility

E	G	S	M	U
	X			

Consider the following factors:
- will it be possible to achieve the objective(s) using this strategy?
- availability of facilities/staff;
- realistic phasing and timetable.

Enter your considerations:

This is an ambitious project but the co-applicants have a strong team and good collaborators. They have a project plan with realistic phasing and are aware of the risks.

2.6 Overall quality assessment

E	G	S	M	U
	X			

Please give reasons for your score:

This is a strong team developing and testing an educational intervention focused on enhancing the clinical practice of district nursing in promoting independence in their patients. It is an ambitious plan culminating in a pilot cluster randomized trial which will provide useful data but not necessarily evidence that points to implementation by others. However findings from this and the other WPs will assist district nurses and district nursing service providers in better understanding how best to support older adults in regaining independence.

3. Budget

Legenda: TH (Too high), R (realistic), TL (too low)

3.1 Budget

TH	R	TL
	X	

Available budget per project is € 600.000,- maximum. Co-financing of at least 25% is required.

Please explain:

I am not able to comment on this aspect . I have ticked realistic as the form would not allow me to proceed without ticking a box.
