# Supplementary material for: Person-centered shared decision-making and data-informed district nursing care to enhance independence: Protocol for a feasibility study
Source: Int J Nurs Stud Adv. 2026 Jun 1;11:100569. doi: 10.1016/j.ijnsa.2026.100569 (PMC13266195; doi:10.1016/j.ijnsa.2026.100569)
Supplement: Supplementary file 9 [file mmc9.pdf]

Subsidieprogramma / Subsidy programme	:	**Verpleging en Verzorging**
Dossiernummer / Dossier number	:	**80-86300-98-057**
Aanvrager / applicant	:	**Prof. dr. B.M. Buurman**
Projecttitel / Project title	:	**Data Driven Essential Care in District Nursing: improving patient outcomes and maintaining independence**
Beoordelingscode / Assessment code	:	**B.2021.003A1**

1. General information

Please read before reviewing the proposal.

Programme

The programme Nursing and Care is designed to enhance the professionalism of nurses, carers and nursing specialists, and is thus important for the attractiveness of the profession and the quality of care.

Call for proposals

The purpose of this call is the acquisition of five projects. These projects should contribute to the evidence base for nursing and/or care practices on the theme of essential care. The results and recommendations that emerge from the studies must have a good likelihood of being applied (in other words, they must be implementable in practice).

2. Criteria

Legenda: E (Excellent), G (Good), S (Sufficient), M (Moderate), U (Unsatisfactory)

2.1 Objective, problem definition and assignment

E	G	S	M	U
	X			

Consider the following factors:

- how clear and specific the objective is;
- how clear and verifiable the problem definition/assignment is and whether it is consistent with the objective, namely substantiating nursing and care activities, better patient outcomes, and improving the quality of care;
- the value added to existing knowledge or practice
- the theoretical or empirical evidence presented in support of the problem definition/assignment

Enter your considerations:

The proposal provides a clearly presented outline of the intended challenges to be addressed and the study objectives. The 5 work packages identified clearly align with the objective and appear achievable int the allotted study period. The need for District Nursing (DN) will rise exponentially over the forthcoming years and maintaining patient independence, avoiding admission to hospital and ensuring that the provision of DN care is data-driven appears appropriate. The objectives and work packages are underpinned by positive patient outcomes. The project will have great national relevance with all DN teams in the Netherlands using Electronic Nursing Records and 80% alignment with the use of the OMAHA system. Such national compliance with an electronic system isn't evident in other countries, but project outcomes may well be a drive for this.
The project is underpinned by contemporary evidence.

2.2 Strategy

E	G	S	M	U
	X			

Consider the following factors:

- clarity;
- adequacy in terms of problem definition/assignment;
- adequacy of chosen methods and analyses including theoretical and/or empirical substantiation;
- a power analysis if applicable:
- collection and analysis of the experiences from patients and their relatives;
- if there is a target group:

- the way in which the strategy reflects the factors gender, age, ethnicity and/or other characteristics relevant to the objective;
- degree of collaboration with intermediate and/or ultimate target group (the patient/client perspective).

Enter your considerations:

The proposal is clearly defined alongside the drivers for this project. Data is recorded across DN teams but there is a lack of a mechanism to embed this, learn from the data and thus improve DN efficiencies, patient care and person-centred outcomes. The application of the Medical Research Council Framework and the Goal-based care framework are innovative approaches that appear appropriate for the project. The project, at all stages, involves patients and caregivers to ensure the suitability of the project and intended outcomes.
The target group is set for older adults, a growing area of need and addresses equality and diversity principles effectively. Patient and carer involvement underpins all 5 work packages.

2.3 Knowledge transfer

E	G	S	M	U
	X			

Consider the following factors:

- nurses and carers are the end users of the results. All knowledge which stems from the research line must be accessible, applicable in practice, be of national relevance and lead to an improved quality of care.
- cooperation with knowledge centres and educational- and practical institutions
- the involvement of patients and/or patient organizations
- the knowledge will be incorporated into training programmes and curricula of educational establishments, in extra training and further education activities and, where possible, in guidelines/standards

Enter your considerations:

This proposal to ensure essential care is data-driven for Dn teams in the Netherlands will empower DN teams, encourage enhanced shared decision making with patients and carers, ensuring care is optimised and independence encouraged. Shared goal setting will require education for nursing teams but steps are planned to integrate the patient's voice into this area of development.
The proposal is clear that learning from the project will be relevant and disseminated nationally (and also internationally). The Netherlands has a high level of compliance with ENR and a single system (80%) - this is not necessarily the case worldwide but project outcomes may influence such developments in the future.
The focus on the older person's independence and shared decision making within DN practice will have demonstrable and evidenced positive patient outcomes.
The inclusion of PhD opportunities for DNs, bachelor student involvement and future training are all included.

2.4 Project group

E	G	S	M	U
X				

Consider the following factors:

- relevant expertise concerning research, practice, education, implementation, and patient perspective;
- familiarity with area in question;
- prior activities and products

Enter your considerations:

The team have the relevant knowledge and skills to undertake this project. They have also developed international relationships in relation to the OMAHA system and the Goals-based care framework. This team is also underpinned by a clear patient involvement principle. Experience appears relevant, especially the inclusion of PhD opportunities for 2 DNs - this will ensure that the project remains aligned to current DN practice.

2.5 Feasibility

E	G	S	M	U
	X			

Consider the following factors:
- will it be possible to achieve the objective(s) using this strategy?
- availability of facilities/staff;
- realistic phasing and timetable.

Enter your considerations:

The 5 work packages and timeline appear appropriate and achievable. Working patterns and the systems already in place will underpin this projects ability to remain within the time scale.
The strategy aligns with the proposed objectives.

2.6 Overall quality assessment

E	G	S	M	U
	X			

Please give reasons for your score:

The overall quality score is good - great national relevance to the project with potential for international dissemination to improve paractice.

3. Budget

Legenda: TH (Too high), R (realistic), TL (too low)

3.1 Budget

TH	R	TL
	X	

Available budget per project is € 600.000,- maximum. Co-financing of at least 25% is required.

Please explain:

The project budget outlined appears appropriate for a project of this size and significance.
Integrated PhD opportunities for 2 DNs are a significant inclusion and appropriate for the focus of the project.
Noted 25% co-funding.
