# Supplementary material for: Person-centered shared decision-making and data-informed district nursing care to enhance independence: Protocol for a feasibility study
Source: Int J Nurs Stud Adv. 2026 Jun 1;11:100569. doi: 10.1016/j.ijnsa.2026.100569 (PMC13266195; doi:10.1016/j.ijnsa.2026.100569)
Supplement: Supplementary file 10 [file mmc10.pdf]

Subsidieprogramma / Subsidy programme	:	**Verpleging en Verzorging**
Dossiernummer / Dossier number	:	**80-86300-98-057**
Aanvrager / applicant	:	**Prof. dr. B.M. Buurman**
Projecttitel / Project title	:	**Data Driven Essential Care in District Nursing: improving patient outcomes and maintaining independence**
Beoordelingscode / Assessment code	:	**B.2021.00391**

1. General information

Please read before reviewing the proposal.

Programme

The programme Nursing and Care is designed to enhance the professionalism of nurses, carers and nursing specialists, and is thus important for the attractiveness of the profession and the quality of care.

Call for proposals

The purpose of this call is the acquisition of five projects. These projects should contribute to the evidence base for nursing and/or care practices on the theme of essential care. The results and recommendations that emerge from the studies must have a good likelihood of being applied (in other words, they must be implementable in practice).

2. Criteria

Legenda: E (Excellent), G (Good), S (Sufficient), M (Moderate), U (Unsatisfactory)

2.1 Objective, problem definition and assignment

E	G	S	M	U
X				

Consider the following factors:

- how clear and specific the objective is;
- how clear and verifiable the problem definition/assignment is and whether it is consistent with the objective, namely substantiating nursing and care activities, better patient outcomes, and improving the quality of care;
- the value added to existing knowledge or practice
- the theoretical or empirical evidence presented in support of the problem definition/assignment

Enter your considerations:

The objectives were very clear and well articulated. The problem was outlined in adequate detail and is innovative in its approach of utilising healthcare data. Excellent added value with its approach and including service users and nurses. Theoretical underpinning was presented and is appropriate. It reads well and the project is logical in its methodology and WPs.

2.2 Strategy

E	G	S	M	U
X				

Consider the following factors:

- clarity;
- adequacy in terms of problem definition/assignment;
- adequacy of chosen methods and analyses including theoretical and/or empirical substantiation;
- a power analysis if applicable:
- collection and analysis of the experiences from patients and their relatives;
- if there is a target group:
    - the way in which the strategy reflects the factors gender, age, ethnicity and/or other characteristics relevant to the objective;
    - degree of collaboration with intermediate and/or ultimate target group (the patient/client perspective).

Enter your considerations:

It is clear and my only comment here is that 'independence' will need to be clearly defined at the start of the project as patients and nurses perceptions on what it is, may be very different! Similarly, shared-decision making must be clearly defined so that common goals and objectives are achieveable. Project shows good level of collaboration and appropriate inclusion.

2.3 Knowledge transfer

E	G	S	M	U
X				

Consider the following factors:

- nurses and carers are the end users of the results. All knowledge which stems from the research line must be accessible, applicable in practice, be of national relevance and lead to an improved quality of care.
- cooperation with knowledge centres and educational- and practical institutions
- the involvement of patients and/or patient organizations
- the knowledge will be incorporated into training programmes and curricula of educational establishments, in extra training and further education activities and, where possible, in guidelines/standards

Enter your considerations:

A nice project in terms of translatable research and inclusion of PhD students who are DNs is a really good inclusion.
It may be worthwhile having some patients on a steering group too.
Train the trainer is excellent but it must be done using a standardised protocol to ensure a homogenous approach in all centres.

2.4 Project group

E	G	S	M	U
X				

Consider the following factors:

- relevant expertise concerning research, practice, education, implementation, and patient perspective;
- familiarity with area in question;
- prior activities and products

Enter your considerations:

A strong research group who have a practical and academic investment in the project. All are appropriate. The importance of IT support and experts in IT for this cannot be under-estimated. Very clear instructions on what is required from IT will be required to ensure the databases anis useful and different systems can 'talk' to each other (always a problem across healthcare).

2.5 Feasibility

E	G	S	M	U
X				

Consider the following factors:

- will it be possible to achieve the objective(s) using this strategy?
- availability of facilities/staff;
- realistic phasing and timetable.

Enter your considerations:

Yes, the project timelines are feasible but the risks are high for each WP to progress, they are dependent on the previous WP being completed. This will need close day to day project management.
My only comment is that when analysing the work of the DNs, it would be really useful to add how much time each task takes and this would help with work allocation (especially as the number of older people increases). Also the acuity of each task should be considered as part of the project and this would be really meaningful data and help with day-to-day workforce planning (often under-estimated and leads to unrealistic expectations).

2.6 Overall quality assessment

E	G	S	M	U
X				

Please give reasons for your score:

A very interesting project and well thought out. Shared decision-making is very important and although seen as a 'soft' approach, it is key on successful implementation of a service such as district nursing. Partnership with patients should be central to care and this project aims to do this. Good luck and I look forward to seeing the

results.
I really like the PhD candidates as part of the study- a great idea.

3. Budget

Legenda: TH (Too high), R (realistic), TL (too low)

3.1 Budget

TH	R	TL
	X	

Available budget per project is € 600.000,- maximum. Co-financing of at least 25% is required.

Please explain:

This project is not cheap but given the work required, I believe it is realistic.
